# Association of asthma severity and educational attainment at age 6–7 years in a birth cohort: population-based record-linkage study

**DOI:** 10.1136/thoraxjnl-2020-215422

**Published:** 2020-11-11

**Authors:** Annette Evans, Daniel Farewell, Joanne Demmler, Amrita Bandyopadhyay, Colin Victor Eric Powell, Shantini Paranjothy

**Affiliations:** 1 Division of Population Medicine, Cardiff University School of Medicine, Cardiff, UK; 2 Centre for the Development and Evaluation of Complex Interventions for Public Health Improvement, Data Science Building, School of Medicine, Swansea University, Swansea, UK; 3 Administrative Data Research Wales, Swansea University Medical School, Swansea, UK; 4 Department of Pediatric Emergency Medicine, Senior Attending Physician (Head of Research), Sidra Medicine, Education City, Al Rayyan, Qatar; 5 Centre for the Development and Evaluation of Complex Interventions for Public Health Improvement, Cardiff University College of Arts Humanities and Social Sciences, Cardiff, UK; 6 Centre for Improvement in Population Health through E-records Research, Institute of Life Science Medical School, Swansea University, Swansea, UK

**Keywords:** asthma epidemiology, asthma, asthma guidelines, asthma in primary care, asthma mechanisms, paediatric asthma, respiratory infection, viral infection

## Abstract

**Background:**

There is conflicting research about the association between asthma and poor educational attainment that may be due to asthma definitions. Our study creates seven categories of current chronic and acute asthma to investigate if there is an association for poorer educational attainment at age 6–7 years, and the role of respiratory infections and school absence.

**Methods:**

This study used a population-based electronic cross-sectional birth cohort 1998–2005, in Wales, UK, using health and education administrative datasets. Current asthma or wheeze categories were developed using clinical management guidelines in general practice (GP) data, acute asthma was inpatient hospital admissions and respiratory infections were the count of GP visits, from birth to age 6–7 years. We used multilevel logistic regression grouped by schools to ascertain if asthma or wheeze was associated with not attaining the expected level in teacher assessment at Key Stage 1 (KS1) adjusting for sociodemographics, perinatal, other respiratory illness and school characteristics. We tested if absence from school was a mediator in this relationship using the difference method.

**Results:**

There were 85 906 children in this population representative cohort with 7-year follow-up. In adjusted multilevel logistic regression, only asthma inpatient hospital admission was associated with increased risk for not attaining the expected level at KS1 (adjusted OR 1.14 95% CI (1.02 to 1.27)). Lower respiratory tract infection (LRTI) GP contacts remained an independent predictor for not attaining the expected level of education. Absence from school was a potential mediator of the association between hospital admission and educational attainment.

**Conclusions:**

Clinicians and educators need to be aware that children who have inpatient hospital admissions for asthma or wheeze, or repeated LRTI, may require additional educational support for their educational outcomes.

**Key messages**

**What is the key question?**

- Is asthma or wheeze severity during the first six years of life associated with educational attainment at age 6–7 years, should we consider the influence of respiratory infections and does school absence explain the relationship?

**What is the bottom line?**

- After multivariable adjustment, asthma severity has no association with education outcomes at age 6–7 years, but inpatient hospital admissions for acute asthma or wheeze were associated with increased risk for not attaining the expected level of education for children. Lower respiratory tract infections were also an independent predictor for not attaining the expected level in education. School absence was found to potentially explain the association between acute asthma and lower educational attainment.

**Why read on?**

- This is the first study to consider interactions between asthma and respiratory infections coded in general practice consultations and hospital admissions on educational attainment. The cohort contained 85 000 children and rich covariate information, allowing us to adjust for numerous factors including sociodemographics, birth and school characteristics in modelling the association between asthma and education outcome.

## Introduction

Asthma is a common childhood condition, with prevalence of current asthma (symptoms within the last year) at age 7 years estimated to be 12% in the UK,^1^ similar to Australia^2^ and the USA.^3^ Cumulative prevalence of wheeze was found to be between 15% and 26% during the first 7 years of childhood in the UK,^4^ with current wheeze at age 6–7 years from 7% in the Indian subcontinent to 21% in English language centres (UK, Australia, Canada, New Zealand) and Oceania (22%).^5^ Healthcare and societal burden of asthma in the UK was thought to be in excess of £1.1 billion in 2011–2012.^6^

Clinicians mostly follow asthma management guidelines^7 8^ that advise step changes in medication or a hospital admittance by age of the child. Decisions to step up or step down in the management plan are based on assessment usually after any hospital admission for acute exacerbation and include consideration of previous hospital admission, Paediatric Intensive Care Unit (PICU) admission, recent steroids, psychological and family issues, compliance/adherence issues, response to initial treatment, distance from home and family preference. Chronic asthma is managed using regular reviews, and guidelines differ for recommendations of a step change due to frequency of exacerbations either to enable no acute exacerbations or when more than two to three exacerbations occur in a year. These guidelines for asthma include school attendance but do not include evidence on the impact of hospital admissions or the burden of co-occurring respiratory infections on educational outcomes.

Asthma aetiology is multifactorial, diagnosed by a combination of symptoms of inflammation in the airways with reversible airway narrowing and airway hyper-responsiveness. Acute asthma exacerbations present as the onset of wheeze and respiratory distress. In young children, clinicians often diagnose viral-induced wheeze but do not diagnose asthma, as symptoms may resolve as the child grows older. Upper respiratory tract infections (URTIs) are also common during childhood, and found to occur between three and eight times a year in primary or preschool children.^9 10^ Children with asthma may experience more severe symptoms when they acquire a respiratory infection.^11^

Children with asthma who suffer frequent exacerbations that may require intervention by a general practitioner or a hospital stay may miss school days, resulting in lower educational attainment compared with their peers, but evidence about asthma between age 5 and 9 years is mixed. Two studies in the USA and UK have reported that well-managed asthma had little impact on educational attainment^12 13^ or for repeating a school year,^14 15^ although others showed those with asthma had lower educational attainment^16 17^ compared with those without. Three studies examined severity of asthma with conflicting outcomes: one study in New Zealand showed no difference in educational outcomes,^17^ two studies associated higher severity with poorer school readiness^18^ or educational attainment.^19^ These conflicting results may be due to different definitions of asthma, reliance on parental reporting, inclusion of older children and teenagers, and not adjusting for important confounders such as birth characteristics, other respiratory illness including common infections or school factors in statistical analyses.

This study investigates whether or not asthma severity is associated with educational attainment using a teacher-based assessment at age 7 years (Key Stage 1, KS1). Our study adds to the evidence because it uses a large population-based cohort, enabling us to derive measures of current acute asthma and categories of chronic asthma severity that closely match current asthma management guidelines using general practice (GP) diagnoses and prescriptions, and hospital admissions administrative data. We explored whether respiratory infections may act as an additional exposure and interact with the association of asthma on educational attainment. We explored the role of school absences where data were available (the school year when KS1 was taken where assessment usually starts in the final 2½ months) in the relationship between asthma severity and educational outcome. Our analysis took account of multiple important confounders (perinatal factors and social deprivation) and school factors not previously seen modelled together in other studies for this specific age group.

## Methods

### Study design and setting

We used a population-based cross-sectional electronic cohort of all children born in Wales, from 1 January 1998 to 31 August 2005. This was a preplanned record-linkage study consistent with the broad aims of the cohort, with linkage to prospectively collected health and education administrative data. Data were extracted for asthma, wheeze and other respiratory diagnosis from GP or inpatient hospital admissions data. Children were followed up from birth to age 6–7 years, to their first teacher-based educational assessment at KS1, through record linkage between education and health routine datasets.

### Data sources and participants

We analysed deidentified data in the Secure Anonymized Information Linkage (SAIL) databank, UK,^20 21^ using the Wales Electronic Cohort for Children (WECC),^22^ developed from five health and demographic national datasets detailed in online supplement table S1. WECC includes all children living in Wales during 1990–2012 in the Wales Demographic Service (WDS), from registration with a GP in the National Health Service (NHS). We record-linked these children to the General Practice Database (GPD), containing all contacts with general medical practitioners (including nurse appointments), and to national educational databases through NHS number (health datasets only), name, date of birth, gender, and phonetic and soundex version of names (online supplement table S1).

10.1136/thoraxjnl-2020-215422.supp1 Supplementary data

**Figure 1:**
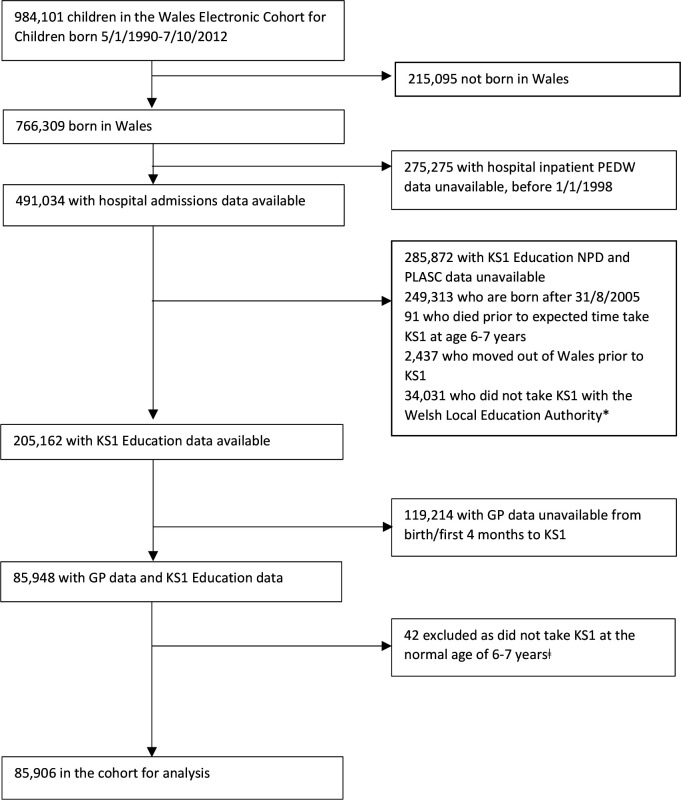
Anonymised participant selection. KS1, Key Stage 1; NPD, National Pupil Database; PEDW, Patient Episode Database Wales; PLASC, Pupil Level Annual School Census. *Private schools, severely disabled children who are not catered for by special educational needs provision in the LEA school system, those outside administrative systems, for example, travellers. ‡To adhere to no overlap between exposure and outcome time windows. LEA, Local Education Authority.

Children were excluded if they were not born in Wales (as these children had high levels of missing data for birth characteristics), born before 1 January 1998 (as electronic GP and hospital inpatient admissions data were not available from the data sources before this date) or after 31 August 2005 (as they would not have sufficient follow-up to age 6–7 years). Further exclusions were children who died or moved out of Wales before age 7 years, who did not attend a local education authority school, did not take KS1 at the normal age or where the child’s GP practice had not signed up to SAIL (figure 1). At the time of data extraction, the GPD in the SAIL databank had over 40% of the 474 practices in Wales signed up, and over 1.9 million people.

### Outcome

The main outcome was attainment of the expected educational level (yes/no), in compulsory assessment in Wales at age 6–7 years (KS1), normally taught between age 5 and 7 years.^23^ KS1 is a teacher assessment of a language, mathematics and science with overall level awarded between 0 and 4, where below level 2 constitutes not attaining the expected level at KS1. Children who were disapplied (not taking the subject), not awarded the level, unable to provide an assessment or working towards the assessment level were coded as did not attain the expected level at KS1 in this study, in agreement with definitions by the Welsh Department of Education.

### Exposures

Current chronic asthma severity was derived using data on diagnoses and prescriptions, and acute asthma from inpatient hospital admissions. Asthma or wheeze was ascertained in GP data (using a set of previously published Read codes in the GPD^6 24^) or International Classification of Diseases 10th Revision (ICD10) codes J45-46, R06.2 in the first coding position of any inpatient hospital admission (including hospital transfers) between birth and age 7 years. We developed two algorithms to derive categorical variables that describe current chronic asthma severity: one excluded wheeze-only diagnoses (called the asthma severity algorithm), while the second, a broader algorithm included wheeze (called the wheeze severity algorithm). Chronic asthma severity was recorded for each year of the child’s life, aligned to five levels of asthma management (based partly on yearly prescriptions) described in the National Heart, Lung and Blood institute (NHLBI) Expert Panel Report 3: Guidelines for the Diagnosis and Management of Asthma 2007 (USA).^7^ An additional category was created where only a diagnosis was recorded. These guidelines were compared with current UK assessment and stepwise treatment guidelines for the clinical management of asthma^8 25^ to create an algorithm that met both criteria, as described in table 1 (coding lists in online supplemental tables 3-6; online supplemental figure S1). These two algorithms enabled us to compare analyses based on definitive diagnoses of asthma (from ongoing prescriptions of inhaled corticosteroid or preventer medication, or tests such as spirometry—usually only achievable from age 5 years) with those in a broader definition, that is, children diagnosed with either wheeze or asthma.

**Table 1:** Description of asthma severity using administrative health datasets

Asthma or wheeze severity algorithms for this analysis (see coding in online supplemental tables 2–6)	
**Asthma severity algorithm**	
**Category**	**Coding description**
None	No diagnosis of asthma between birth and KS1 (age 6–7 years)
Diagnosis only	Ever had an asthma diagnosis in either GP or hospital inpatient admissions data* between birth and age at KS1 (age 6–7 years)
Intermittent bronchodilator	>1 and ≤12 prescriptions of asthma bronchodilator including nebulisers in any 1 year of life, and ever had an asthma diagnosis in either GP or hospital inpatient admissions data* between birth and age at KS1 (age 6–7 years)
Persistent mild	Inhaled corticosteroid including nebulised or >12 bronchodilator prescriptions in any 1 year of life†, and ever had an asthma diagnosis in either GP or hospital inpatient admissions data* between birth and age at KS1 (age 6–7 years)
Persistent moderate	Inhaled corticosteroid including nebulised or >12 bronchodilator prescriptions in any 1 year of life†, and at least one prescription of a preventer medication (eg, long-acting beta agonists), and ever had an asthma diagnosis in either GP or hospital inpatient admissions data* between birth and age at KS1 (age 6–7 years)
Persistent severe	Asthma injection prescription, immunosuppressant therapy prescription or >3 oral corticosteroid prescriptions in any 1 year of life, and ever had an asthma diagnosis in either GP or hospital inpatient admissions data* between birth and age at KS1 (age 6–7 years)
**Wheeze severity algorithm**	
‘No diagnosis of asthma or wheeze’ replaces ‘no diagnosis of asthma’, and ‘ever had a wheeze or asthma diagnosis’ replaces ‘ever had an asthma diagnosis’ in the definitions for the asthma severity algorithm above	

*Inpatient hospital diagnosis is the first diagnosis code of first consultant episode of each person’s continuous stay in hospital including transfers.

†In this cohort, no child had >12 bronchodilator prescriptions in any 1 year of his/her life.

GP, general practice; KS1, Key Stage 1.

For the main analysis, we chose the most severe category of asthma from the yearly calculations as exposure status for each child between birth and age 7 years for simplicity, and in further analyses, split status by age less than 2 years, 2–<5 years, 5–<7 years to closely match the guidelines and age when KS1 is taught. We labelled the current chronic asthma severity categories as ‘none’, ‘diagnosis only’, ‘intermittent bronchodilator’, ‘persistent mild’, persistent moderate’ and ‘persistent severe’ to match guideline categories (table 1).

Two further variables were created for acute asthma or wheeze using inpatient hospital admissions in the Patient Episode Database Wales dataset from birth to KS1. Acute asthma was defined with ICD10 codes J45-46, and acute asthma or wheeze included an additional code R06.2 for wheezing. Where children did not have a previous diagnosis, the initial hospital admission was excluded from these variables to allow for the possibility that prescribed medication following this admission may control symptoms.

Respiratory illness was ascertained from GP data and categorised as follows: lower respiratory tract infection (LRTI) including bronchiolitis when bronchitis was also coded, upper respiratory tract infection (URTI), influenza or pneumonia, bronchiolitis, chronic upper respiratory disease, chronic lower respiratory disease, croup and unspecified respiratory illness (online supplemental table S6). GP contacts between birth and KS1 were categorised by frequency, for example, 0, 1, 2 or more, to give a measure of burden of disease. For URTI, GP contacts were grouped into 0, 1–4, 5–6, 7+ as they were more common.

### Potential confounders, covariates and effect modifiers

Birth characteristics were available from WECC for sex, gestation at birth, small for gestational birth (<10th centile) adjusted for gestation and sex, parity, major or minor congenital anomaly, maternal age, breast feeding recorded at birth or at 6–8 weeks, maternal cigarette smoking in first trimester, academic season of birth, and urban or rural dwelling at birth. Deprivation at birth was categorised into quintiles using Townsend deprivation scores of small area of residence Lower Super Output Area (LSOA) from the 2001 census. School datasets provided variables on school attended at KS1 assessment, school moves, year took KS1, percentage of absence from school (in the school year when KS1 was taken where assessment usually starts in the final 2½ months) and free school meals eligibility in the school year take KS1 (used as a proxy for deprivation level beyond birth).

### Statistical analyses

**Figure 2:**
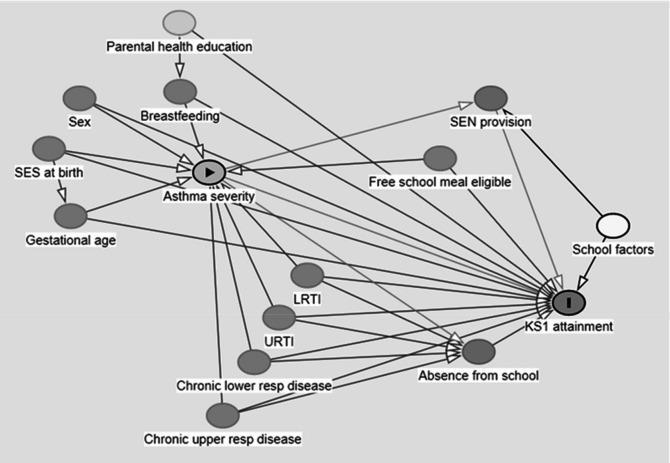
Directed acyclic graph causal diagram of asthma severity and Key Stage 1 attainment (at age 6–7 years). Chronic lower resp disease, chronic lower respiratory disease; chronic upper resp disease, chronic upper respiratory disease; KS1, Key Stage 1; LRTI, lower respiratory tract infection; SES, socioeconomic status (Townsend deprivation quintile, maternal cigarette smoking in the first trimester), gestational age (at birth), asthma severity (general practice contacts and hospital inpatient admissions), breastfeeding (at birth or 6–8 weeks); SEN, special educational needs, school factors (academic season of birth, school moves, urban or rural area at birth, schools level variable); URTI, upper respiratory tract infection. All theoretical causal relationships are drawn, where relationships may be in both directions over time, the dominant theoretical relationship is chosen.

The variable selection for analyses was informed by using a directed acyclic graph (figure 2).^26^ Models were adjusted for school factors^27^ because of the known association with variability in educational attainment. Special educational needs (SEN) provision at school was excluded from models due to its potential partial causal pathway between asthma and educational attainment (possibly occurring due to disruption to daily activities and missed school days), to investigate the impact of asthma rather than asthma after the effects of SEN provision. Likelihood ratio tests were used to investigate two-way interactions between chronic asthma severity and acute asthma, between these asthma variables and respiratory infections, sex or deprivation. We tested if absence from school was a mediator between asthma and educational attainment using the difference method.^28^

Data were analysed using Stata V.13, hypothesis tests were two-sided and statistical significance was set at p<0.05. The clinical significance of asthma may prevent a child from attaining the expected level at KS1, which may lead to reduced life chances in later education and employment, with little comparative cost to update pre-existing guidance for asthma for clinicians and educators. Multilevel logistic regression (QR decomposition) was used to obtain ORs for not attaining the expected level at KS1. To account for the possibility of unobserved shared factors leading to correlation of educational outcomes within schools, we used school-level random effects. We used Hosmer-Lemeshow goodness of model fit tests. Missing data were imputed using multiple imputations by chained equations of all the variables in the model^29^ (five imputations), and no interpretable difference was found between imputed models and models with no-answer categories included.

Modelling was repeated for asthma and wheeze exposure split by age group for pairs of current chronic severity and acute variables to prevent possible effects of collinearity between different age groups.

Sensitivity analysis was performed by including only one randomly selected child from each mother, to mitigate the potential for spuriously small SEs arising from correlation between outcomes for children born to the same mother.

## Results

There were 85 906 children born in Wales between 1998 and 2005 with 7-year follow-up (figure 1). Baseline characteristics (table 2) were similar to those of the general population of Wales (online supplemental table S2). The prevalence of current chronic asthma (using the asthma severity algorithm) in this cohort between birth and KS1 (age 6–7 years) was 12.5%, with 0.6% categorised as diagnosis only, 2.7% with intermittent bronchodilator, 7.3% persistent mild, 1.6% persistent moderate, 0.3% persistent severe, and for acute asthma 4.1% had an inpatient hospital admission (table 3). For the wider definition that included wheeze (wheeze severity algorithm), the prevalence was 21.4%, with noticeably higher proportions in categories of diagnosis only, intermittent bronchodilator, persistent mild, and acute asthma or wheeze inpatient hospital admissions (table 3). As expected for children with asthma, there were higher proportions in males, lower gestational age at birth, no breast feeding, maternal smoking in the first trimester, higher deprivation and urban compared with rural dwelling at birth (table 2).

**Table 2:** Demographics of the study population

	Asthma severity algorithm				Acute asthma
	No asthma	Diagnosis only or intermittent bronchodilator	Persistent mild*	Persistent moderate or severe	Inpatient hospital admission†
N	75 156	2839	6247	1664	3487
Sex=male (%)	37 906 (50)	1624 (57)	3767 (60)	1026 (62)	2284 (66)
Gestation at birth‡					
≤32	990 (1)	73 (3)	180 (3)	56 (3)	152 (4)
33–36	3974 (5)	173 (6)	390 (6)	131 (8)	245 (7)
37+ weeks	65 870 (88)	2429 (86)	5339 (86)	1389 (84)	2913 (84)
Small for gestational birth (<10th centile)‡=yes (%)	6474 (9)	272 (10)	578 (9)	141 (9)	337 (10)
Parity ≥1	43 284 (58)	1564 (55)	3572 (57)	947 (57)	2079 (60)
Congenital anomaly§=Yes (%)	3516 (5)	187 (7)	373 (6)	115 (7)	273 (8)
Maternal age at childbirth					
<18 (%)	1956 (3)	119 (4)	163 (3)	37 (2)	125 (4)
18–24 (%)	18 713 (25)	878 (31)	1857 (30)	502 (30)	1126 (32)
25–29 years (%)	21 072 (28)	802 (28)	1776 (28)	470 (28)	947 (27)
30–34 (%)	21 750 (29)	696 (25)	1622 (26)	434 (26)	868 (25)
35+ (%)	11 627 (16)	343 (12)	826 (13)	219 (13)	420 (12)
Breast feeding¶					
No (%)	28 242 (38)	1138 (40)	2602 (42)	716 (43)	1540 (44)
Yes (%)	31 996 (43)	1054 (37)	2282 (37)	661 (40)	1318 (38)
NA (%)	14 918 (20)	647 (23)	1363 (22)	287 (17)	629 (18)
Maternal smoking in first trimester					
No (%)	18 493 (25)	622 (22)	1351 (22)	382 (23)	729 (21)
Yes (%)	5448 (7)	243 (9)	491 (8)	130 (8)	308 (9)
NA (%)	51 215 (68)	1974 (70)	4405 (70)	1152 (69)	2450 (70)
Townsend deprivation quintile at birth					
1—least (%)	13 723 (18)	424 (15)	920 (15)	218 (13)	460 (13)
2 (%)	14 745 (20)	466 (16)	1102 (18)	307 (18)	593 (17)
3 (%)	15 171 (20)	561 (20)	1244 (20)	340 (20)	676 (19)
4 (%)	15 414 (21)	609 (22)	1414 (23)	390 (23)	824 (24)
5—most (%)	15 884 (21)	768 (27)	1547 (25)	403 (24)	921 (26)
Free school meals eligible**=yes (%)	12 299 (16)	643 (23)	1270 (20)	338 (20)	796 (23)
School absence percentage††					
<5 (%)	19 867 (48)	608 (41)	1254 (39)	297 (31)	624 (33)
5–9 (%)	12 776 (31)	476 (32)	1162 (36)	332 (35)	679 (36)
10–14 (%)	4473 (11)	196 (13)	462 (14)	174 (18)	300 (16)
15–19 (%)	1533 (4)	75 (5)	141 (4)	70 (7)	117 (6)
20+ (%)	922 (2)	60 (4)	111 (3)	44 (5)	92 (5)
NA (%)	1479 (4)	73 (5)	117 (4)	38 (4)	83 (4)

*Inhaled corticosteroid or alternative.

†Excludes first admission if before first GP visit.

‡6% missing data evenly found across asthma groups.

§Major or minor.

¶At birth or 6–8 weeks.

**In Key Stage 1 (KS1), assessment year as a proxy for level of deprivation beyond birth.

††In school year when KS1 was taken where assessment usually starts in the final 2½ months, subsample due to availability of school absence data, births between September 2000 and August 2004, N=46 470.

**Table 3:** Asthma severity algorithms and acute asthma

	Asthma severity algorithm* n (%)	Acute asthma (hospital inpatient admission)‡ n (%)	Wheeze severity algorithm† n (%)	Acute asthma or wheeze (hospital inpatient admission)‡ n (%)
N	85 906	3487 (4.1)	85 906	4668 (5.4)
None	75 156 (87.5)	0 (0)	67 508 (78.6)	0 (0)
Diagnosis only	532 (0.6)	169 (32)	2237 (2.6)	683 (30.5)
Intermittent bronchodilator	2307 (2.7)	474 (21)	6465 (7.5)	938 (14.5)
Persistent mild	6247 (7.3)	1978 (32)	7964 (9.3)	2164 (27.2)
Persistent moderate	1365 (1.6)	661 (48)	1407 (1.6)	673 (47.8)
Persistent severe	299 (0.3)	205 (69)	325 (0.4)	210 (64.6)

*Developed with an asthma diagnosis.

†Developed with either a wheeze or asthma diagnosis.

‡Excludes first admission if before first general practice visit.

Over 20% of children in each asthma severity category had an asthma inpatient hospital admission, with 69% of children in the persistent severe category admitted; patterns were relatively similar for asthma or wheeze severity (table 3). Multiple GP contacts for a child were found for respiratory infections, 11% of children had seven or more GP contacts for URTI and 8% had three or more GP contacts for LRTI.

Children with higher asthma severity had more GP contacts for LRTI, 17% of children with intermittent bronchodilator and 50% with persistent severe asthma had three or more GP contacts for LRTI, compared with 6% for those without a diagnosis of asthma (table 4). GP contacts for seven or more URTIs were 17% and 32% for the same asthma severity categories, compared with 10% in children without an asthma diagnosis. Similar proportions across the categories were seen for the broader wheeze severity algorithm (data not shown). Of the 8% of children with three or more LRTI GP contacts, 31% also had seven or more URTI GP contacts, and greater numbers were found in the most deprived quintile and those eligible for free school meals (data not shown).

**Table 4:** Respiratory illness between birth and before Key Stage 1 assessment by asthma severity

	Asthma severity algorithm						Acute asthma
	No asthma	Diagnosis only	Intermittent bronchodilator	Persistent mild*	Persistent moderate	Persistent severe	Inpatient hospital admission†
N	75 156	532	2307	6247	1365	299	3487
LRTI GP contacts§							
0 (%)	52 170 (69)	346 (65)	1071 (46)	2515 (40)	445 (33)	71 (24)	1196 (34)
1 (%)	13 361 (18)	94 (18)	520 (23)	1443 (23)	291 (21)	50 (17)	765 (22)
2 (%)	5336 (7)	48 (9)	319 (14)	863 (14)	207 (15)	30 (10)	511 (15)
3+ (%)	4289 (6)	44 (8)	397 (17)	1426 (23)	422 (31)	148 (50)	1015 (29)
URTI GP contacts							
0 (%)	20 266 (27)	204 (38)	427 (19)	1022 (16)	169 (12)	25 (8)	598 (17)
1–4 (%)	39 817 (53)	243 (46)	1195 (52)	3167 (51)	614 (45)	126 (42)	1663 (48)
5–6 (%)	7190 (10)	39 (7)	301 (13)	855 (14)	204 (15)	52 (17)	472 (14)
7+ (%)	7683 (10)	46 (9)	384 (17)	1203 (19)	378 (28)	96 (32)	754 (22)
GP contacts							
Influenza and pneumonia 1+ (%)	2067 (3)	20 (4)	82 (4)	304 (5)	113 (8)	16 (5)	242 (7)
Bronchiolitis 1+ (%)	3063 (4)	30 (6)	221 (10)	662 (11)	189 (14)	54 (18)	538 (15)
Chronic lower respiratory disease 1+ (%)	465 (1)	6 (1)	28 (1)	96 (2)	32 (2)	6 (2)	89 (3)
Unspecified respiratory illness 1+ (%)	392 (1)	<5 (0)	24 (1)	39 (1)	15(1)	<5 (1–2)	20 (1)
Chronic upper respiratory disease GP contacts							
0 (%)	69 704 (93)	490 (92)	2006 (87)	5250 (84)	1050 (77)	244 (82)	2907 (83)
1 (%)	4192 (6)	31 (6)	207 (9)	699 (11)	203 (15)	40 (13)	418 (12)
2+ (%)	1260 (2)	11 (2)	94 (4)	298 (5)	112 (8)	15 (5)	162 (5)
Croup GP contacts¶							
0 (%)	69 722 (93)	500 (94)	2077 (90)	5543 (89)	1146 (84)	239 (80)	3091 (89)
1+ (%)	5434 (7)	32 (6)	230 (10)	704 (11)	219 (16)	60 (20)	396 (11)

*Inhaled corticosteroid or alternative.

†Excludes first admission if before first GP visit.

‡

§Includes bronchiolitis if coded with bronchitis.

¶Original categories 0, 1, 2+ changed due to small numbers concerning anonymity rules.

GP, general practice; URTI, upper respiratory tract infection.

Within the cohort, 14 935 (17%) children did not attain the expected level at KS1 (table 5), average age at assessment was 7 years 1 month (SD 3.5 months). Unadjusted analysis showed increased risk for all categories in the asthma severity algorithm for non-attainment of the expected level at KS1, the highest were diagnosis only (OR 1.58 95% CI 1.29 to 1.95), with similar size ORs for persistent severe and acute asthma from inpatient hospital admission. Over time, there were increased numbers of children who became diagnosed with persistent mild or persistent moderate asthma (online supplemental figure S2).

**Table 5:** Multilevel multivariable models of asthma severity algorithm, respiratory illness and not attaining the expected level at Key Stage 1 (at 6–7 years)

	Not attained/Total (%)	Unadjusted OR (95% CI)	Asthma severity algorithm Multivariable* OR (95% CI)	Wheeze severity algorithm Multivariable* OR (95% CI)
N	14 935/85 906 (17)			
Asthma severity algorithm				
None (ref)	12 733/75 156 (17)	Ref	Ref	NA
Diagnosis only	134/532 (25)	1.58 (1.29 to 1.95)	1.21 (0.97 to 1.52)	NA
Intermittent bronchodilator	444/2307 (19)	1.11 (1.00 to 1.24)	0.90 (0.79 to 1.01)	NA
Persistent mild	1260/6247 (20)	1.21 (1.13 to 1.30)	0.97 (0.89 to 1.05)	NA
Persistent moderate	298/1365 (22)	1.36 (1.18 to 1.55)	1.06 (0.91 to 1.24)	NA
Persistent severe	66/299 (22)	1.45 (1.09 to 1.93)	1.02 (0.75 to 1.40)	NA
Hospital inpatient admission (acute asthma)†=yes (%)	837/3487 (24)	1.48 (1.36 to 1.61)	1.14 (1.02 to 1.27)	NA
Wheeze severity algorithm				
None (ref)	11 252/67 508 (17)	Ref	NA	Ref
Diagnosis only	451/2237 (20)	1.24 (1.11 to 1.39)	NA	1.05 (0.94 to 1.19)
Intermittent bronchodilator	1271/6465 (20)	1.20 (1.12 to 1.28)	NA	1.01 (0.94 to 1.08)
Persistent mild	1582/7964 (20)	1.22 (1.14 to 1.29)	NA	0.97 (0.90 to 1.04)
Persistent moderate	306/1407 (22)	1.37 (1.20 to 1.57)	NA	1.06 (0.91 to 1.23)
Persistent severe	73/325 (23)	1.53 (1.16 to 2.00)	NA	1.08 (0.80 to 1.45)
Hospital inpatient admission (acute asthma or wheeze)†=yes (%)	1112/4668 (24)	1.48 (1.37 to 1.59)	NA	1.14 (1.04 to 1.25)
LRTI‡ GP contacts§ (ref=none)				
1	2729/15 759 (17)	1.05 (1.00 to 1.10)	1.01 (0.96 to 1.07)	1.01 (0.96 to 1.06)
2	1260/6803 (18)	1.14 (1.07 to 1.22)	1.05 (0.97 to 1.13)	1.04 (0.97 to 1.12)
3+	1421/6726 (21)	1.33 (1.25 to 1.43)	1.15 (1.06 to 1.24)	1.14 (1.06 to 1.23)
URTI¶ GP contacts (ref=none)				
1–4	7727/45 162 (17)	0.98 (0.93 to 1.02)	1.00 (0.95 to 1.05)	1.00 (0.95 to 1.05)
5–6	1483/8641 (17)	1.00 (0.93 to 1.07)	1.01 (0.94 to 1.09)	1.01 (0.94 to 1.09)
7+	1842/9790 (19)	1.10 (1.03 to 1.17)	1.08 (1.01 to 1.16)	1.08 (1.00 to 1.16)
GP contacts				
Influenza and pneumonia 1+ (ref=None)	436/2602 (17)	0.96 (0.86 to 1.07)	NA	NA
Bronchiolitis 1+ (ref=none)	884/4219 (21)	1.26 (1.16 to 1.36)	1.01 (0.93 to 1.10)	1.00 (0.92 to 1.09)
Chronic lower respiratory disease 1+ (ref=none)	156/633 (25)	1.39 (1.15 to 1.69)	1.18 (0.96 to 1.45)	1.18 (0.96 to 1.45)
Unspecified respiratory illness 1+ (ref=none)	69/475 (15)	0.75 (0.57 to 0.98)	NA	NA
Chronic upper respiratory disease GP contacts (ref=none)				
1	914/5372 (17)	0.98 (0.91 to 1.06)	NA	NA
2+	308/1790 (17)	1.02 (0.90 to 1.16)	NA	NA
Croup GP contacts (ref=none)				
1	884/5247 (17)	0.98 (0.91 to 1.06)	NA	NA
2+	264/1432 (18)	1.14 (0.99 to 1.31)	NA	NA
Townsend deprivation quintile				
1—least (ref)	1517/15 285 (10)	ref	Ref	Ref
2	2271/16 620 (14)	1.27 (1.18 to 1.37)	1.16 (1.07 to 1.25)	1.16 (1.07 to 1.25)
3	2944/17 316 (17)	1.53 (1.42 to 1.65)	1.28 (1.18 to 1.38)	1.28 (1.18 to 1.38)
4	3494/17 827 (20)	1.84 (1.71 to 1.98)	1.42 (1.32 to 1.54)	1.42 (1.32 to 1.54)
5—most	4668/18 602 (25)	2.32 (2.16 to 2.49)	1.54 (1.42 to 1.67)	1.54 (1.42 to 1.67)

*Adjusted for all variables in the table significant at the 5% level in unadjusted analyses, sex, gestation at birth, small for gestational age (<10th centile), parity, major or minor congenital anomalies, maternal age (25–29 years, <18, 18–24, 30–34, 35+), breast feeding at birth or 6–8 weeks, maternal smoking in first trimester, free school meals in school year take KS1 assessment (to approximate deprivation beyond birth), academic season of birth (autumn, spring, summer), school moves from start school to KS1 (1+), urban or rural (inc. town) dwelling at birth, year take Key Stage 1 (ref 2010).

†Excludes first admission if before first GP visit.

‡Lower respiratory tract infection.

§Includes bronchiolitis if coded with bronchitis.

¶Upper respiratory tract infection.

GP, general practice; LRTI, lower respiratory tract infection.

Following adjustment for social deprivation, birth and school characteristics, only asthma inpatient hospital admission remained associated with increased risk for not attaining the expected level at KS1 (aOR 1.14 95% CI (1.02 to 1.27)). Presentations to primary care for respiratory tract infections (RTIs) were also independently associated with increased risk for not attaining the expected level at KS1, aOR 1.15 95% CI (1.06 to 1.24) for three or more presentations for LRTI, and aOR 1.08 95% CI (1.01 to 1.16) for seven or more URTI GP contacts (table 5).

There was no evidence for interactions between the asthma severity algorithm GP categories or asthma inpatient hospital admissions, or between the asthma severity variables and other respiratory illness including LRTI and URTI, sex of the child or deprivation at the 5% level of significance. The increased risk in unadjusted ORs in the asthma severity algorithm categories, fell to a third of their size when inpatient hospital admission was added to the model, and then became non-significant at the 5% level when a measure of deprivation, gender or LRTI was added to the model. Model goodness of fit tests found interactions between confounders were only adjustments to main effects (in opposing directions if significant at p<0.05 with no monotonic pattern) and therefore were not included in the final model interpretation (data not shown).

Very similar results were found for the wheeze severity algorithm and non-attainment of the expected level at KS1 (table 5).

Further analyses using asthma variables divided into age groups found no association for current chronic asthma severity, but higher odds for not attaining the expected level at KS1 for three or more inpatient hospital admissions aOR 1.4 95% CI (1.0 to 2.0) in children age 2–<5 years, with similar results for 0–<2 years and 5–<7 years. For wheeze or asthma, only three or more inpatient hospital admissions from age 2 years onwards had an increased association for not attaining the expected level at KS1 (online supplemental table S7).

A subsample analysis of births from 1 September 2000 to 31 August 2004 (where school absence data were available) showed after adjustment for school absence asthma inpatient hospital admissions were no longer associated with risk for not attaining the expected level at KS1 (aOR 1.05 (95% CI 0.90 to 1.24), but LRTI remained an independent predictor (aOR 1.13 (95% CI 1.02 to 1.26)) (online supplemental table S8). Similar results were found for not attaining the expected level at KS1 for acute asthma or wheeze when absence from school was added to the model.

Models with only one child per mother in the cohort showed no substantial difference to our main results, indicating that there was no problematic underestimation of variances arising through correlation between mothers.

## Discussion

Our results show that children with an inpatient hospital admission for asthma was associated with an increased risk for not attaining the expected level at KS1 at age 6–7 years after controlling for current asthma severity, deprivation, birth characteristics, other respiratory illness and school characteristics. Very similar results were obtained using a broader definition of asthma which included wheeze. Presentations to primary care for respiratory infections, particularly LRTI, were independently associated with not attaining the expected level at KS1, even after adjustment for school absence. We found no interaction between asthma and LRTI, or asthma and URTI for educational attainment outcome. We found school absence in the year a child takes KS1 assessment may be a potential mediator in the association between asthma hospital admissions and not attaining the expected level at KS1.

This is the first study that explores the association of asthma and common respiratory ailments on educational attainment in childhood. The findings suggest that inpatient hospital admissions for asthma and recurring respiratory illness from birth to KS1 can have long-term effects on a child through their educational attainment.

The use of a second algorithm that included wheeze allowed us to investigate potentially under-reported asthma, particularly relevant to children under 5 years. It highlights that children treated for only wheeze through hospital admission were associated with increased risk for not attaining the expected level at KS1.

In previous research, GP data and parental surveys recording GP diagnosis of asthma^1–3^ in the UK, USA and Australia show similar results for this age group, reassuring us of the validity and classification of asthma in our study. For children with wheeze or asthma diagnosis, our UK prevalence matches a global survey,^5^ but is slightly lower than other UK surveys,^4 30^ most likely due to differences in cohort demographics.

In previous cohorts or surveys in population representative studies adjusted for confounding from deprivation (or proxy such as maternal education), a detrimental association of current asthma (defined as with a prescription) at age 6 years was found for reading (aOR 2.20 95% CI (1.08 to 4.51))^17^ (measured as ≥6 months behind), but with no difference in mathematics in New Zealand. In the USA, asthma defined as requiring attention or treatment in the last year and limiting a child’s activity found children less likely to be school ready, but no difference in language skills compared with children without asthma.^18^ Another study found no significant difference (p>0.05) for children age 5–7 years in parental reporting for repeating a school year.^14^ None of these studies separated chronic asthma severity and acute asthma or adjusted for the full range of respiratory illness, school or birth characteristics (the latter study only adjusting for birth weight).

Other cohorts that adjusted for deprivation used wider age bands (children 5–16 years), found no association^12 13^ with asthma, possibly due to adjustment for SEN provision,^13^ or concluded that lower marks were explained by higher days absent.^16^ A Canadian study found children with severe asthma (defined as using asthma medication) was associated with poorer mathematics scores but not in reading.^19^ None of these studies adjusted for birth characteristics or other respiratory illnesses and our smaller adjusted risks demonstrate the importance of controlling for confounding. At key developmental ages, younger children may have greater risk of hospital admission for asthma or wheeze as they experience potentially more associated RTIs at younger ages,^30^ and may be less able to communicate symptoms or manage their condition.

Studies measuring absence from school as an outcome^31^ or in addition to educational attainment^12 16 17 19^ mostly reported 2 more days absence in a year compared with children without asthma, and higher absence for younger children.^32^ When more severe asthma was measured using reliever prescriptions or emergency department visits, children had 7 or more days absence from school in a year,^19 31^ and agree with the higher proportions of absence we found across our categories for children with asthma compared with those without (table 2).

Consistent with other studies,^13 33^ we found higher prevalence of asthma in children living in more deprived areas, possibly due to poorer housing conditions,^33^ environmental factors or less healthy diets^5^ that could also lead to more RTIs.

### Strengths

Clinical diagnosis in our algorithms mean records of medications that sometimes have other uses, a bronchodilator medication trial over 6 weeks or reversibility test at the clinician appointment are most likely excluded. General practitioners have indicated that symptoms used to classify severity of asthma or wheeze may not be fully recorded during GP consultations but inform prescribing, therefore using prescriptions should minimise any selection or ascertainment bias.

The use of routine data from clinical practitioners on diagnoses and prescriptions rather than parental reports removes the potential impact of recall bias.

### Limitations

Hospital admission policies may differ across Wales, but testing models with local education authorities (the same geographical areas as health boards before 2009) shows little correlation and minimal selection bias. Some families contact their GP more often than others; but any additional visits to the GP would bias risk for not attaining the expected level at KS1 towards the null (more visits would be required to show a significant association). Children in more deprived areas may be less compliant to asthma management plans; we found no effect modification between chronic asthma severity or acute asthma and our measures of deprivation for educational attainment, residual confounding may remain, but would only highlight that barriers to health still exist.

This study does not include accident and emergency admissions data, and may underestimate the number of attendances for acute asthma exacerbations; an audit showed 86% of exacerbations were treated in GP in 1991.^34^

Our study only investigated period prevalence; a recent paper investigating worsening asthma in the previous year found an association with poorer educational attainment.^35^

This study uses a large population-based representative cohort with asthma or wheeze severity algorithms akin to asthma management guidelines found in the USA and UK. Only 5% of children move out of Wales each year, and GP data included were found to represent the Welsh population so results should be generalisable in the UK and other countries with similar sociodemographic and health systems.

### Implications for research and practice

Clinicians and educators need to be aware that children who have inpatient hospital admissions for asthma or wheeze, or repeated LRTI GP visits, may need additional educational support for their educational outcomes. For children with asthma, the association between LRTI and not attaining the expected level at KS1 was not above that expected for children without asthma, but more children with asthma had multiple GP visits for LRTI (indicating accumulating risk).
