# Correction: *Association of asthma severity and educational attainment at age 6–7 years in a birth cohort: population-based record-linkage study*

**DOI:** 10.1136/thoraxjnl-2020-215422corr1

**Published:** 2022-02-17

Evans A, Farewell D, Demmler J, *et al*. Association of asthma severity and educational attainment at age 6–7 years in a birth cohort: population-based record-linkage study. *Thorax* 2021;76:116–125

In the interests of transparency, we, the authors, bring attention to two data issues in the results due to coding error. The interpretation of the results of this paper have not changed. We report the omission of 805 children from the cohort of 85 906 children (<1%) where 26 children had died and 779 children had moved out of Wales between the ages of 6–15 years. The death or move from Wales occurred after the outcome of interest: Key Stage 1 (KS1) at age 6–7 years. Of those children omitted who died, low numbers were classified with persistent moderate asthma or wheeze and none were classified with persistent severe asthma or wheeze. We have rerun the analyses and find only up to a 1% difference between the two population-based cohorts in cross-tabulations and no difference in percentages to one decimal place for the developed asthma or wheeze severity categories or hospital admissions (table 3). In modelling we found there was up to a 0.02 difference between the population-based cohorts in the ORs in adjusted and unadjusted variables reported except for Townsend deprivation at birth where the difference in ORs ranged from least deprived of 0.02 to most deprived 0.08. In the omitted children who died, 81% had no asthma in either inpatient hospital admissions or General Practice (GP) data, and 69% had no wheeze or lower respiratory tract infection (LRTI) in GP data (or inpatient hospital admissions for wheeze). Rates of no asthma, wheeze and LRTI were the same as the population-based cohort for children omitted who had moved out of Wales. In addition, in the supplementary table S7 (subgroup analysis split by age of child) we realise that a hospital admission that occurred before the child’s first GP visit for either asthma or wheeze was not excluded from this data and admissions under age 1 year were excluded. We submit a correction for this table. For subgroups 2-<5 years and 5-<7 years we report only differences of up to 2% between the two population-based cohorts and up to a 0.1 difference in adjusted odds ratios (reported to one decimal place) for asthma or wheeze severity or number of hospital admissions associated with not attaining KS1. In children age 0-<2 years we find a difference of up to 3% and up to 0.4 difference in adjusted ORs but interpretation of the data has not changed. For results of children age 2-<5 years quoted in the main paper we find a point estimate increase of 0.1 for three or more asthma inpatient hospital admissions but no change in the 95% CI in adjusted models for not attaining the expected level at KS1. We have made the updated data set available for access in the SAIL databank as required.

**Difference between cohorts=**805 children

Original cohort in the paper=85 906 children

New cohort including children who died after KS1 or moved out of Wales in cohort follow-up timeframe=86 711 children

Extra children in new cohort who died after KS1 in cohort follow-up timeframe=26 children

Extra children in new cohort who moved out of Wales after KS1 in cohort follow-up timeframe =779 children

**Difference between cohorts – age at death**

**Table:** 

Age at death	Count
6–8	6
9–11	8
12–15	12
Total	26

**Difference between cohorts – moved out of Wales**

**Table:** 

Age when moved out of wales	Count
7	182
8	166
9	127
10	125
11	84
12	45
13	32
14	10
Total	779

**Table 2:** Demographics of the study population (Bold numbers show differences in this table compared with the original published journal article)

	Asthma algorithm				
	No asthma	Diagnosis only or intermittent bronchodilator	Persistent mild*	Persistent moderate or severe	Inpatient hospital admission†
N	**75 870**	**2872**	**6298**	**1671**	**3513**
Gestation at birth‡					
≤32	**1001** (1)	73 (3)	**183** (3)	56 (3)	**154** (4)
33–36	**4010** (5)	**177** (6)	**393** (6)	131 (8)	**247** (7)
37+weeks	66 473 (88)	**2457** (86)	**5379** (**85**)	**1393** (**83**)	**2933** (84)
Congenital anomaly§=Yes(%)	**3552** (5)	187 (7)	**376** (6)	115 (7)	**274** (8)
Townsend deprivation quintile at birth					
1 - least (%)	13 853 (18)	**426** (15)	**922** (15)	**220** (13)	**462** (13)
2 (%)	14 905 (20)	**477** (**17**)	**1112** (18)	**307** (18)	**598** (17)
3 (%)	15 291 (20)	**566** (20)	**1257** (20)	**341** (20)	**683** (19)
4 (%)	15 564 (21)	**618** (22)	**1421** (23)	**393** (**24**)	**829** (24)
5 - most (%)	16 035 (21)	**774** (27)	**1565** (25)	**404** (24)	**927** (26)
Free school meals eligible¶=yes(%)	12 469 (16)	**652** (23)	**1285** (20)	**343** (**21**)	**805** (23)
School absence percentage¶**					
<5 (%)	19 986 (48)	**613** (41)	**1261** (39)	**298** (31)	**627** (33)
5–9 (%)	12 892 (31)	**478** (32)	**1171** (36)	**334** (35)	**684** (36)
10–14 (%)	**4515** (11)	**198** (13)	**467** (14)	**175** (18)	**304** (16)
15–19 (%)	**1558** (4)	**78** (5)	**143** (4)	**70** (7)	**120** (6)
20+ (%)	**927** (2)	60 (4)	111 (3)	**46** (5)	**93** (5)
NA (%)	**1528** (4)	**75** (5)	**118** (4)	38 (4)	**84** (4)

*Inhaled corticosteroid or alternative.

†For asthma or wheeze.

‡6% missing data evenly found across asthma groups.

§Major or minor.

¶In year preceding Key Stage one assessment proxy start date first May.

**Sub-sample due to availability of school absence data, births between Sept 2000-Aug 2004 n=47 140.

**Table 3:** Asthma severity algorithms (Bold numbers show differences in this table compared with the original published journal article)

	Asthma severity algorithm* N (%)	Wheeze severity algorithm† N (%)
N	**86 711**	**86 711**
No asthma	**75 870** (87.5)	**68 143** (78.6)
Diagnosis only	**539** (0.6)	**2263** (2.6)
Intermittent Bronchodilator	**2333** (2.7)	**6535** (7.5)
Persistent Mild	**6298** (7.3)	**8031** (9.3)
Persistent moderate	**1372** (1.6)	**1414** (1.6)
Persistent severe	299 (0.3)	325 (0.4)
Hospital inpatient admission‡=yes(%)	**3513** (4.1)	**4715** (5.4)

*developed with an asthma diagnosis

†developed with either a wheeze or asthma diagnosis

‡excludes first admission if before first GP visit

**Table 4:** Respiratory illness between birth and before Key Stage one assessment by asthma severity (Bold numbers show differences in this table compared with the original published journal article)

	Asthma severity algorithm						
	No asthma	Diagnosis only	Intermittent bronchodilator	Persistent mild*	Persistent moderate	Persistent severe	Inpatient hospital admission†
N	**75 870**	**539**	**2333**	**6298**	**1372**	299	**3513**
Hospital inpatient admission (Algorithm 1)‡=yes (%)	0	173 (32)	480 (21)	1993 (32)	662 (48)	205 (69)	3513 (100)
LRTI GP contacts§							
0 (%)	**52 711 (70)**	**353 (66)**	**1084 (47)**	**2540** (40)	**448** (33)	71 (24)	**1211 (35)**
1 (%)	**13 473** (18)	**94 (17)**	**524** (23)	**1453** (23)	**292** (21)	50 (17)	**769** (22)
2 (%)	**5365** (7)	48 (9)	**325** (14)	**870** (14)	**209** (15)	30 (10)	**514** (15)
3+ (%)	**4321** (6)	44 (8)	**400** (17)	**1435** (23)	**423** (31)	148 (50)	**1019** (29)
URTI GP contacts							
0 (%)	**20 781** (27)	**207** (38)	**430 (18)**	**1030** (16)	169 (12)	25 (8)	**602** (17)
1–4 (%)	**40 135** (53)	**247** (46)	**1213** (52)	**3195** (51)	**619** (45)	126 (42)	**1679** (48)
5–6 (%)	**7238** (10)	39 (7)	**302** (13)	**865** (14)	**206** (15)	52 (17)	**475** (14)
7+ (%)	**7716** (10)	46 (9)	**388** (17)	**1208** (19)	**378** (28)	96 (32)	**757** (22)

*Inhaled corticosteroid or alternative.

†For asthma or wheeze.

‡Excludes first admission if before first GP visit.

§includes bronchiolitis if coded with bronchitis.

**Table 5:** Multilevel multivariable models of asthma severity algorithm, respiratory illness and not attaining Key Stage 1 (at 6–7 years) (Bold numbers show differences in this table compared with the original published journal article)

	Not attained / Total (%)	Univariable OR (95% CI)	Asthma severity algorithm 1 Multivariable* OR (95% CI) OR (95% CI)	Wheeze severity algorithm 2 Multivariable* OR (95% CI)
N	**15115/86 711** (17)			
Asthma severity algorithm				
No asthma (ref)	**12892/75 870** (17)	Ref	Ref	NA
Diagnosis only	**135/539** (25)	**1.57 (1.28 to 1.93**)	**1.22 (0.98 to 1.52**)	NA
Intermittent bronchodilator	**451/2333** (19)	1.11 (1.00 to 1.24)	**0.90** (**0.80** to 1.01)	NA
Persistent Mild	**1270/6298** (20)	1.21 (1.13 to **1.29**)	0.96 (**0.89** to 1.04)	NA
Persistent moderate	**301/1372** (22)	1.36 (**1.19** to 1.55)	1.05 (0.90 to **1.23**)	NA
Persistent severe	66/299 (22)	1.45 (1.09 to **1.92**)	1.02 (0.75 to 1.40)	NA
Hospital inpatient admission (asthma severity algorithm)†=yes(%)	**846/3513** (24)	1.48 (1.36 to 1.61)	**1.15 (1.03 to 1.28**)	NA
Wheeze severity algorithm				
No asthma (ref)	**11388/68 143** (17)	Ref	NA	ref
Diagnosis only	**457/2263** (20)	1.24 (1.11 to 1.39)	NA	1.06 (0.94 to 1.19)
Intermittent bronchodilator	**1291/6535** (20)	1.20 (1.12 to 1.28)	NA	1.01 (0.94 to **1.09**)
Persistent mild	**1597/8031** (20)	**1.21** (1.14 to 1.29)	NA	**0.97** (0.90 to **1.04**)
Persistent moderate	**309/1414** (22)	**1.38** (1.20 to 1.57)	NA	**1.06** (0.91 to **1.23**)
Persistent severe	73/325 (23)	**1.52** (1.16 to 2.00)	NA	1.08 (**0.81** to 1.45)
Hospital inpatient admission (wheeze severity algorithm)†=yes(%)	**1129/4715** (24)	1.48 (**1.38** to 1.59)	NA	1.14 (1.04 to 1.25)
LRTI‡ GP contacts§ (ref=None)				
1	**2758/15 886** (17)	1.05 (1.00 to 1.10)	1.01 (0.96 to **1.07**)	1.01 (**0.96** to 1.06)
2	**1270/6847 (19**)	1.14 (**1.06** to 1.22)	1.05 (**0.98** to 1.13)	**1.05 (0.98 to 1.13**)
3+	**1431/6771** (21)	1.33 (**1.24 to 1.42**)	**1.15 (1.07 to 1.24**)	**1.15 (1.06 to 1.23**)
URTI¶ GP contacts (ref=None)				
1–4	**7809/45 535** (17)	0.98 (0.93 to 1.02)	1.00 (**0.96** to 1.05)	1.00 (**0.96** to 1.05)
5–6	**1496/8702** (17)	**0.99** (0.93 to 1.07)	**1.02 (0.95 to 1.10**)	1.01 (**0.95 to 1.10**)
7+	**1853/9832** (19)	**1.09** (**1.02** to 1.17)	**1.09** (1.01 to **1.17**)	1.08 (1.00 to 1.16)
GP contacts				
Influenza and pneumonia 1+ (ref=None)	**441/2616** (17)	0.96 (0.86 to 1.07)	NA	NA
Bronchiolitis 1+ (ref=None)	**896/4258** (21)	1.26 **(1.17 to 1.37**)	**1.02 (0.94 to 1.11**)	**1.01 (0.93 to 1.10**)
Chronic lower respiratory disease 1+ (ref=None)	**158/635** (25)	**1.41 (1.16 to 1.70**)	**1.16 (0.95 to 1.42**)	**1.16 (0.95 to 1.42**)
Respiratory unknown 1+ (ref=None)	**70/477** (15)	**0.76 (0.58 to 1.00**)	NA	NA
Chronic upper respiratory disease GP contacts (ref=None)				
1	**924/5417** (17)	0.98 (0.91 to 1.06)	NA	NA
2+	**310/1799** (17)	1.02 (0.90 to 1.16)	NA	NA
Croup GP contacts (ref=None)				
1	**890/5284** (17)	0.98 (0.91 to 1.06)	NA	NA
2+	**266/1439** (19)	1.14 (0.99 to 1.31)	NA	NA
Townsend deprivation quintile				
1 - least (ref)	**1533/15 421** (10)	ref	Ref	Ref
2	**2307/16 801** (14)	**1.28** (1.18 to **1.38**)	**1.18 (1.09 to 1.28**)	**1.18 (1.09 to 1.27**)
3	**2980/17 455** (17)	**1.54 (1.43 to 1.66**)	**1.32 (1.22 to 1.43**)	**1.32 (1.22 to 1.43**)
4	**3523/17 996** (20)	1.84 (1.71 to 1.98)	**1.47 (1.36 to 1.58**)	**1.47 (1.36 to 1.58**)
5 – most	**4730/18 778** (25)	2.32 (2.16 to 2.49)	**1.63 (1.51 to 1.76**)	**1.63 (1.51 to 1.76**)

*adjusted for all variables in the table significant at the 5% level in univariable analyses, sex, gestation at birth, small for gestational age (<10^th^ centile), parity, major or minor congenital anomalies, maternal age (25–29 years,<18, 18–24, 30–34, 35+), breastfeeding at birth or 6–8 weeks, maternal smoking in first trimester, free school meals eligible in year preceding Key Stage one assessment proxy start date first May to approximate deprivation beyond birth, academic season of birth (autumn, spring, summer), school moves from start school to KS1 (1+), urban or rural (inc. town) dwelling at birth, year take Key Stage 1 (ref 2010).

†Excludes first admission if before first GP visit; c Lower respiratory tract infection.

‡Lower respiratory tract infection.

§Includes bronchiolitis if coded with bronchitis.

¶Upper respiratory tract infection.

**Table:** 

		Difference between cohorts			
		Died between age 6 and 15 years n=26		Moved out of wales between age 6 and 15 years n=779	
		Count	Column N %	Count	Column N %
Attained expected level at KS1	Yes	14	53.8%	611	78.4%
	Not attained	12	46.2%	168	21.6%
	Total	26	100.0%	779	100.0%

**Figure 1:**
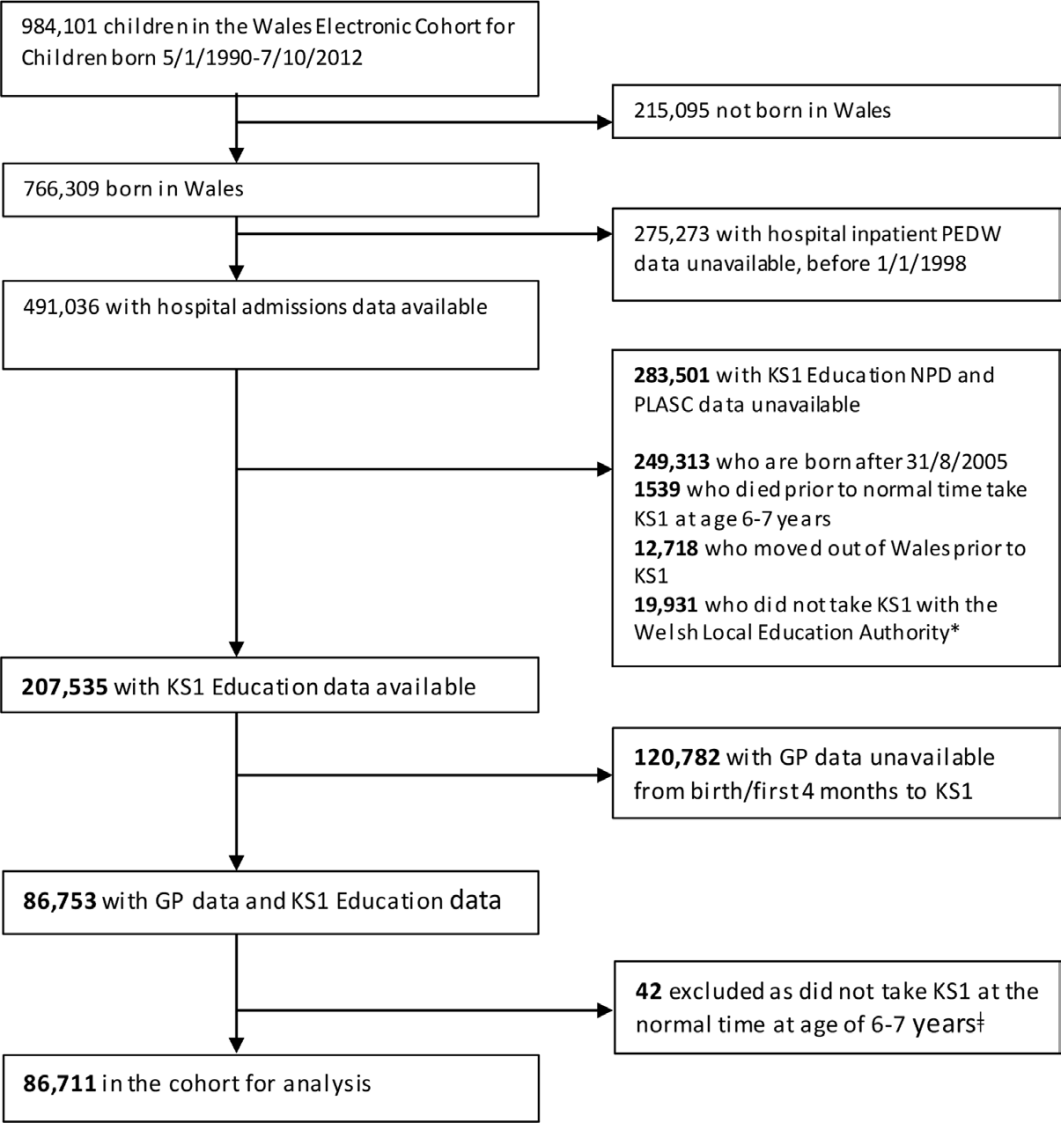
Anonymised participant selection (Bold numbers show differences in this table compared with the original published journal article). PEDW, Patient Episode Database Wales; KS1, Key Stage 1; NPD, National Pupil Database; PLASC, Pupil Level Annual School Census. *private schools, severely disabled children who are not catered for by Special Educational Needs provision in the LEA school system, those outside administrative systems for example, travellers; ǂ to adhere to no overlap between exposure and outcome time windows.

Updated table from the original cohort in the published journal article with the corrected variables where hospital admission prior to first GP visit is removed, hospital admissions for under 1 year are included. Bold numbers show differences in this table compared with the original published journal paper.

Table S7 Multilevel multivariable models of asthma severity algorithm and asthma inpatient hospital admissions for different ages of the child and not attaining the expected level at Key Stage 1 (at 6–7 years) – repeated for wheeze severity algorithm and wheeze inpatient hospital admissions.

**Table:** 

	**Child age 0 -<2 years**			**Child age 2 -<5 years**			**Child age 5 -<7 years**		
	**Not attained / Total (%)**	**Unadjusted** **OR (95% CI)**	Multivariable* **OR (95% CI)**	**Not attained / Total (%)**	**Unadjusted** **OR (95% CI)**	**Multivariable** OR (95% CI)*	**Not attained / Total (%)**	**Unadjusted OR (95% CI)**	Multivariable* **OR (95% CI)**
N	14935/85 906 (17)								
Asthma severity algorithm									
No asthma (ref)	13459/79 301 (17)	Ref	ref	12980/76 324 (17)	ref	ref	13120/77 041 (17)	ref	ref
Diagnosis only	178/732 (24)	1.5 (1.2 to 1.8)	1.1 (0.9 to 1.3)	133/590 (23)	1.4 (1.1 to 1.7)	1.0 (0.8 to 1.3)	122/664 (18)	1.0 (0.9 to 1.3)	0.8 (0.7 to 1.0)
Intermittent bronchodilator	606/2837 (21)	1.3 (1.2 to 1.4)	1.0 (0.9 to 1.1)	460/2448 (19)	1.1 (1.0 to 1.2)	0.9 (0.8 to 1.0)	330/1715 (19)	1.1 (1.0 to 1.3)	0.9 (0.8 to 1.1)
Persistent Mild	663/2910 (21)	1.4 (1.3 to 1.6)	1.1 (1.0 to 1.2)	1110/5459 (20)	1.2 (1.2 to 1.3)	1.0 (0.9 to 1.1)	1100/5290 (21)	1.3 (1.2 to 1.4)	1.0 (0.9 to 1.1)
Persistent moderate	17/73 (23)	1.6 (0.9 to 2.8)	1.1 (0.6 to 2.0)	213/897 (24)	1.5 (1.3 to 1.7)	1.1 (0.9 to 1.3)	229/1055 (22)	1.3 (1.2 to 1.6)	1.1 (0.9 to 1.3)
Persistent severe	12/53 (23)	1.7 (0.9 to 3.3)	1.3 (0.7 to 2.7)	39/188 (21)	1.3 (0.9 to 1.9)	0.9 (0.6 to 1.3)	34/141 (24)	1.6 (1.1 to 2.4)	1.1 (0.7 to 1.6)
Hospital inpatient admission (asthma severity algorithm)									
1 (%) (ref=0)	**139/518 (27**)	**1.7 (1.4 to 2.1**)	1.1 **(0.9 to 1.4**)	**293/1208** (24)	**1.5 (1.3 to 1.7**)	1.1 (**1.0** to 1.3)	**240/924** (26)	1.6 **(1.4 to 1.9**)	1.3 (1.1 to **1.6**)
2 (%)	**33/103** (32)	**2.0 (1.3 to 3.2**)	**1.2 (0.7 to 1.9**)	**91/331 (28**)	**1.8 (1.4 to 2.3**)	**1.4** (1.1 to **1.9**)	**40/142 (28**)	1.6 (1.1 to 2.4)	1.3 (0.9 to 2.0)
3+ (%)	**20/51 (39**)	**3.1 (1.7 to 5.5**)	**1.8 (1.0 to 3.4**)	66/**221 (30**)	**2.0 (1.5 to 2.7**)	**1.5** (1.0 to 2.0)	**21/67 (31**)	**2.2** (1.3 to **3.8**)	**1.3 (0.7 to 2.4**)
N	14935/85 906 (17)								
Wheeze severity algorithm									
No asthma (ref)	12354/73 804 (17)	ref	Ref	12147/71 977 (17)	ref	ref	12586/74 350 (17)	ref	ref
Diagnosis only	475/2284 (21)	1.3 (1.1 to 1.4)	**1.0** (0.9 to 1.2)	245/1188 (21)	1.2 (1.1 to 1.4)	**1.0** (0.9 to 1.2)	175/915 (19)	1.1 (0.9 to 1.3)	0.9 (0.8 to 1.1)
Intermittent bronchodilator	1206/5738 (21)	1.3 (1.2 to 1.4)	1.0 (1.0 to 1.1)	946/4891 (19)	1.1 (1.1 to 1.2)	1.0 (0.9 to 1.1)	607/3039 (20)	1.2 (1.1 to 1.3)	1.0 (0.9 to 1.1)
Persistent Mild	866/3939 (22)	1.4 (1.3 to 1.5)	1.1 (1.0 to 1.2)	1337/6728 (20)	1.2 (1.1 to 1.3)	1.0 (0.9 to 1.1)	1296/6374 (20)	1.2 (1.2 to 1.3)	1.0 (0.9 to 1.1)
Persistent moderate	19/78 (24)	1.8 (1.0 to 3.0)	**1.2** (0.7 to 2.2)	218/921 (24)	1.5 (1.3 to 1.8)	**1.0** (0.9 to 1.3)	234/1078 (22)	1.4 (1.2 to 1.6)	1.0 (0.9 to 1.2)
Persistent severe	15/63 (24)	1.8 (1.0 to 3.3)	1.4 **(0.7 to 2.6**)	42/201 (21)	1.3 (0.9 to 1.9)	0.9 (0.6 to 1.4)	37/150 (25)	1.7 (1.1 to 2.4)	1.0 (0.7 to 1.7)
Hospital inpatient admission (wheeze severity algorithm)									
1 (%) (ref=0)	**376/1559 (24**)	**1.5 (1.3 to 1.7**)	**1.1 (0.9 to 1.2**)	**372/1655 (23**)	**1.4** (1.2 to 1.5)	1.1 (0.9 to 1.2)	**272/1054 (26**)	1.6 **(1.4 to 1.9**)	1.3 (1.1 to **1.6**)
2 (%)	**115/391 (29**)	**1.9 (1.5 to 2.4**)	**1.2 (0.9 to 1.6**)	**113/501** (23)	**1.3** (1.1 to 1.7)	**1.1** (0.9 to **1.4**)	**49/166** (30)	1.8 (1.2 to 2.5)	1.4 (1.0 to **2.1**)
3+ (%)	**64/215 (30**)	**1.8 (1.4 to 2.5**)	**1.0** (0.7 to **1.4**)	**96/323 (30**)	**1.9 (1.5 to 2.5**)	1.4 **(1.1 to 1.4**)	**23/72 (32**)	**2.3 (1.4 to 3.9**)	**1.5 (0.8 to 2.6**)

*adjusted for sex, gestation at birth, small for gestational age (<10^th^ centile), parity, major or minor congenital anomalies, maternal age (25–29 years,<18, 18–24, 30–34, 35+), breastfeeding at birth or 6–8 weeks, maternal smoking in first trimester, free school meals eligible in year preceding Key Stage one assessment proxy start date first May to approximate deprivation beyond birth, academic season of birth (autumn, spring, summer), school moves from start school to KS1 (1+), urban or rural (inc. town) dwelling at birth, year take Key Stage 1 (ref 2010), other respiratory illness (as described in table 5), Townsend deprivation quintiles; for each age group, asthma severity and hospital admission variables in the table added as a pair to the model without other age group variable pairs due to multicollinearity.

Updated table with update to cohort to include extra 805 children (26 that died age 6–15 years and 779 that moved out of Wales age 6–15 years), with corrected variables where hospital admission prior to the first GP visit has been removed and hospital admissions under 1 year are included.

Table S7 Multilevel multivariable models of asthma severity algorithm and asthma inpatient hospital admissions for different ages of the child and not attaining the expected level at Key Stage 1 (at 6–7 years) – repeated for wheeze severity algorithm and wheeze inpatient hospital admissions.

**Table:** 

	**Child age 0 -<2 years**			**Child age 2 -<5 years**			**Child age 5 -<7 years**		
	**Not attained / Total (%)**	**Unadjusted** **OR (95% CI)**	Multivariable* **OR (95% CI)**	**Not attained / Total (%)**	**Unadjusted** **OR (95% CI)**	Multivariable* **OR (95% CI)**	**Not attained / Total (%)**	**Unadjusted OR (95% CI)**	Multivariable* **OR (95% CI)**
N	15115/86 711 (17)								
Asthma severity algorithm									
No asthma (ref)	13625/80 055 (17)	Ref	ref	13143/77 050 (17)	ref	ref	13283/77 778 (17)041 (17)	ref	ref
Diagnosis only	181/739 (25)	1.5 (1.2 to 1.8)	1.1 (0.9 to 1.3)	135/601 (22)	1.4 (1.1 to 1.7)	1.0 (0.8 to 1.3)	124/672 (18)	1.0 (0.9 to 1.3)	0.8 (0.7 to 1.0)
Intermittent bronchodilator	612/2859 (21)	1.3 (1.2 to 1.4)	1.0 (0.9 to 1.1)	465/2471 (19)	1.1 (1.0 to 1.2)	0.9 (0.8 to 1.0)	332/1729 (19)	1.1 (1.0 to 1.2)	0.9 (0.8 to 1.1)
Persistent Mild	668/2932 (23)	1.4 (1.3 to 1.5)	1.1 (1.0 to 1.2)	1119/5501 (20)	1.2 (1.1 to 1.3)	1.0 (0.9 to 1.1)	1111/5332 (21)	1.3 (1.2 to 1.4)	1.0 (0.9 to 1.1)
Persistent moderate	17/73 (23)	1.6 (0.9 to 2.8)	1.1 (0.6 to 1.9)	214/900 (24)	1.5 (1.3 to 1.7)	1.1 (0.9 to 1.3)	231/1059 (22)	1.3 (1.2 to 1.6)	1.1 (0.9 to 1.3)
Persistent severe	12/53 (23)	1.7 (0.9 to 3.2)	1.3 (0.7 to 2.7)	39/188 (21)	1.3 (0.9 to 1.9)	0.9 (0.6 to 1.3)	34/141 (24)	1.6 (1.1 to 2.4)	1.1 (0.7 to 1.6)
Hospital inpatient admission (asthma severity algorithm)									
1 (%) (ref=0)	139/519 (27)	1.7 (1.4 to 2.1)	1.1 (0.9 to 1.4)	298/1220 (24)	1.5 (1.3 to 1.7)	1.1 (1.0 to 1.3)	241/930 (26)	1.6 (1.4 to 1.9)	1.3 (1.1 to 1.6)
2 (%)	34/104 (33)	2.1 (1.3 to 3.2)	1.2 (0.8 to 1.9)	91/333 (27)	1.7 (1.4 to 2.2)	1.4 (1.1 to 1.9)	40/143 (28)	1.6 (1.1 to 2.4)	1.3 (0.8 to 1.9)
3+ (%)	20/51 (39)	3.1 (1.7 to 5.5)	1.8 (1.0 to 3.5)	67/225 (30)	2.0 (1.5 to 2.7)	1.4 (1.0 to 2.0)	21/68 (31)	2.1 (1.2 to 3.7)	1.3 (0.7 to 2.3)
N	15115/86 711 (17)								
Wheeze severity algorithm									
No asthma (ref)	12503/74 502 (17)	ref	Ref	12300/75 663 (17)	ref	ref	12742/75 069 (17)	Ref	ref
Diagnosis only	484/2309 (21)	1.3 (1.2 to 1.4)	1.0 (0.9 to 1.2)	250/1207 (21)	1.3 (1.1 to 1.5)	1.0 (0.9 to 1.2)	178/925 (19)	1.1 (0.9 to 1.3)	0.9 (0.8 to 1.1)
Intermittent bronchodilator	1220/5787 (21)	1.3 (1.2 to 1.4)	1.0 (1.0 to 1.1)	956/4939 (19)	1.1 (1.1 to 1.2)	1.0 (0.9 to 1.1)	613/3064 (20)	1.2 (1.1 to 1.3)	1.0 (0.9 to 1.1)
Persistent Mild	874/3972 (22)	1.4 (1.3 to 1.5)	1.1 (1.0 to 1.2)	1348/6777 (20)	1.2 (1.1 to 1.3)	1.0 (0.9 to 1.1)	1309/6421 (20)	1.2 (1.2 to 1.3)	1.0 (0.9 to 1.1)
Persistent moderate	19/78 (24)	1.8 (1.0 to 3.0)	1.2 (0.7 to 2.1)	219/924 (24)	1.5 (1.3 to 1.7)	1.1 (0.9 to 1.3)	236/1082 (22)	1.4 (1.2 to 1.6)	1.1 (0.9 to 1.2)
Persistent severe	15/63 (24)	1.8 (1.0 to 3.3)	1.4 (0.7 to 2.6)	42/201 (21)	1.3 (0.9 to 1.9)	0.9 (0.6 to 1.4)	37/150 (25)	1.7 (1.1 to 2.4)	1.1 (0.7 to 1.7)
Hospital inpatient admission (wheeze severity algorithm)									
1 (%) (ref=0)	384/1576 (24)	1.5 (1.3 to 1.7)	1.1 (0.9 to 1.2)	378/1673 (23)	1.4 (1.2 to 1.5)	1.1 (0.9 to 1.2)	273/1061 (26)	1.6 (1.4 to 1.8)	1.3 (1.1 to 1.6)
2 (%)	116/393 (30)	1.9 (1.5 to 2.4)	1.2 (1.0 to 1.6)	113/503 (22)	1.3 (1.1 to 1.7)	1.1 (0.9 to 1.4)	49/167 (29)	1.8 (1.2 to 2.5)	1.4 (1.0 to 2.1)
3+ (%)	65/218 (30)	1.8 (1.4 to 2.5)	1.0 (0.7 to 1.4)	97/328 (30)	1.9 (1.5 to 2.4)	1.4 (1.1 to 1.9)	23/73 (32)	2.2 (1.3 to 3.8)	1.4 (0.8 to 2.5)

*adjusted for sex, gestation at birth, small for gestational age (<10^th^ centile), parity, major or minor congenital anomalies, maternal age (25–29 years,<18, 18–24, 30–34, 35+), breastfeeding at birth or 6–8 weeks, maternal smoking in first trimester, free school meals eligible in year preceding Key Stage one assessment proxy start date first May to approximate deprivation beyond birth, academic season of birth (autumn, spring, summer), school moves from start school to KS1 (1+), urban or rural (inc. town) dwelling at birth, year take Key Stage 1 (ref 2010), other respiratory illness (as described in table 5), Townsend deprivation quintiles; for each age group, asthma severity and hospital admission variables in the table added as a pair to the model without other age group variable pairs due to multicollinearity.

