# Supplementary material for: Association of asthma severity and educational attainment at age 6–7 years in a birth cohort: population-based record-linkage study
Source: Thorax. 2020 Nov 11;76(2):116–25. doi: 10.1136/thoraxjnl-2020-215422 (PMC7815901; doi:10.1136/thoraxjnl-2020-215422)
Supplement: Supplementary data [file thoraxjnl-2020-215422supp001.pdf]

Online Supplement: Association of asthma severity and educational attainment at age 6-7 years in a birth cohort: Population based record-linkage

Table S1: Wales Electronic Cohort for Children (WECC) and linked data sources with descriptions

Table S2: Social-demographic data for Wales

Table S3: General Practice diagnosis codes for Asthma and Wheeze

Table S4: General Practice prescription codes for Asthma

Table S5: General Practice prescription codes for Endocrine corticosteroids

Table S6: General Practice respiratory diagnoses

Figure S1: Changes in asthma prescriptions during the cohort by year take KS1 assessment

Figure S2: Changes in asthma or wheeze severity for discrete years of the child from birth.

Table S7: Multilevel multivariable models of asthma severity algorithm and asthma inpatient hospital admissions for different ages of the child and not attaining the expected level at Key Stage 1 (at 6-7 years) – repeated for wheeze severity algorithm and wheeze inpatient hospital admissions.

Table S8: Sub-sample multilevel multivariable models of asthma severity, acute asthma, respiratory illness and not attaining the expected level at Key Stage 1 (at 6-7 years) adjusted for absence from school in year take KS1 assessment (Week of birth 1 September 2000 to 31 August 2004), N=46,673.

Evans A, *et al. Thorax* 2021; 76:116–125. doi: 10.1136/thoraxjnl-2020-215422

Table S1: Wales Electronic Cohort for Children (WECC) and linked data sources with descriptions

Datasets[a]	Data source
Wales Electronic Cohort for Children	Wales Demographic Service<br>National Community and Child Health database<br>Office of National Statistics birth records<br>Office of National Statistics death records<br>Congenital Anomaly Register and Information Service
Hospital inpatient admission data	Patient Episode Dataset for Wales
General Practice data	General Practice Database
Education data	Pupil Annual School Census<br>National Pupil Database

Data source	Description
Wales Demographic service (WDS)	Register of all people in Wales who have contact with the National Health Service. Information mainly comes from a person registering with a General Practitioner Practice.
National Community Child Health Database (NCCHD) - from 1987	A national database of all children resident in Wales or born in a Welsh hospital, containing data collected at birth such as parity, mode of delivery, gestation, birth weight, gender, breastfeeding, and Apgar Score
Public Health Birth files from the Office for National Statistics (ONSB) - from 2003	Data on all births in Wales or to mothers who are usually resident in Wales
Public Health Mortality Files from the Office for National Statistics (ONSM) - from 2002	Data on all deaths in Wales or of individuals who are usually resident in Wales
Patient Episode Dataset for Wales (PEDW) - from 1998	Demographic and clinical data on all inpatient and day-case admissions in National Health Service Wales hospitals and all Welsh residents treated in other UK countries
Congenital Anomaly Register and Information Service (CARIS) - from 1998	A population-based register of any foetus or infant who has a congenital anomaly whose mother is usually resident in Wales at the time of birth; congenital anomalies are defined by the European network of population-based registries for the epidemiologic surveillance of congenital anomalies

[a] Datasets were linked for each child's record in the SAIL linkage system using an anonymised linking field based on their NHS number (deterministic linkage), name, date of birth, gender and phonetic and soundex versions of names (probabilistic linkage), produced by the NHS Wales Informatics Service, a NHS trusted third party, with more than 99.85% accuracy[21].

Evans A, *et al. Thorax* 2021; 76:116–125. doi: 10.1136/thoraxjnl-2020-215422

Table S2: Social-demographic data for Wales

	Total n	(%)
Townsend Deprivation quintile: from 2003, child's age 0 – 14		
1 - least deprived	-	(19.3)
2	-	(19.3)
3	-	(19.3)
4	-	(20.5)
5 - most deprived	-	(21.7)
Sex: from 2001, child's age 0 – 14 years old[b]		
Male	281767	(51.3)
Female	267437	(48.7)
Breastfeeding at birth: Welsh residents 2011[c]		
No	14469	(40.5)
Yes	18062	(50.6)
no answer	3151	(8.8)
Maternal age at childbirth: Welsh residents 2011 [c]		
<16	57	(0.2)
16-19	2409	(6.8)
19-24	8115	(22.7)
25-29 years	10268	(28.8)
30-34	9107	(25.5)
35+	5722	(16.0)
no answer	4	(0.01)
Gestational age at birth: Welsh residents 2011 [c]		
20-<32 weeks	443	(1.2)
32-<37 weeks	2094	(5.9)
37-43 weeks	32985	(92.4)
no answer	160	(0.4)
Birthweight: Welsh residents 2011 [c]		
Low: < 2500g	2403	(6.7)
Normal: ≥ 2500 - < 4000g	28991	(81.2)
High: ≥ 4000g	4249	(11.9)
no answer	39	(0.1)

[a] Deprivation and health – report for the National Public Health Service for Wales 2004; [b] Welsh data from the UK Census 2001 at https://statswales.gov.wales/; [c] Births in Wales 2001 - 2011: Data from the National Community Child Health Database 2012.

Evans A, *et al. Thorax* 2021; 76:116–125. doi: 10.1136/thoraxjnl-2020-215422

Table S3: General Practice diagnosis codes for Asthma and Wheeze

General Practice Read codes v2 for Asthma Diagnosis and Wheeze symptoms (includes any diagnosis, symptom or procedure codes, extracted 2017)			
**Asthma diagnosis**			
**Code**	**Description**	**Code**	**Description**
H3120	Chronic asthmatic bronchitis	663P2	Asthma limits activit most day
H33..	Asthma	663Q.	Asthma not limiting activities
H330.	Extrinsic (atopic) asthma	663q.	Asthma daytime symptoms
H3300	Extrinsic asthma - no status	663r.	Asthma night symp 1-2 per mth
H3301	Extrinsic asthma + status	663s.	Asthma never causes day symps
H330z	Extrinsic asthma NOS	663t.	Asthma day symp 1-2 per mth
H331.	Intrinsic asthma	663U.	Asthma management plan given
H3310	Intrinsic asthma - no status	663u.	Asthma day symp 1-2 per week
H3311	Intrinsic asthma + status	663V.	Asthma severity
H331z	Intrinsic asthma NOS	663v.	Asthma daytime symps most days
H332.	Mixed asthma	663V0	Occasional asthma
H333.	Acute exacerbation of asthma	663V1	Mild asthma
H334.	Brittle asthma	663V2	Moderate asthma
H335.	Chron asthm w fix airflw obstr	663V3	Severe asthma
H33z.	Asthma unspecified	663W.	Asthma prophylaxis used
H33z0	Status asthmaticus NOS	663x.	Asthma limits walking on flat
H33z1	Asthma attack	66Y5.	Change in asthma managemt plan
H33z2	Late-onset asthma	66Y9.	Step up chnge asthm managmt pl
H33zz	Asthma NOS	66YA.	Step down chnge asthm managmt pl
H35y6	Sequoiosis (red-cedar asthma)	66YC.	Absent work/schl due to asthma
H35y7	Wood asthma	66YE.	Asthma monitoring due
H3B..	Asthma-COPD overlap syndrome	66YJ.	Asthma annual review
H47y0	Detergent asthma	66YK.	Asthma follow-up
663e0	Asthma sometime restr exercise	66YP.	Asthma night-time symptoms
1780	Aspirin induced asthma	66Yp.	Asthma review RCP 3 questions
1781	Asthma trigger - pollen	66YQ.	Asthma monitoring by nurse
1782	Asthma trigger - tobacco smoke	66Yq.	Asthma night symptom 1 to 2 wk
1783	Asthma trigger - warm air	66YR.	Asthma monitoring by doctor
1784	Asthma trigger - emotion	66Yr.	Asthma cause sympt most nights
1785	Asthma trigger - damp	66Ys.	Asthma never caus night symptm
1786	Asthma trigger - animals	66Yu.	Num dy abs sch asthma pst 6 mn
1787	Asthma trigger - seasonal	66YZ.	Does not have asthma man plan
1788	Asthma trigger - cold air	66Yz0	Asthma managemt plan declined
1789	Asthma trigger respiratory inf	66Yz5	Telehealth asthma monitoring
8791	Further asthma - drug prevent.	679J.	Health education - asthma
8793	Asthma control step 0	679J0	Heath educ - asthm self manag
8794	Asthma control step 1	679J1	Heal educ - struct asthma disc
8795	Asthma control step 2	8B3j.	Asthma medication review
8796	Asthma control step 3	8CE2.	Asthma leaflet given
8797	Asthma control step 4	8CMA0	Pat writt asthma pers act plan
8798	Asthma control step 5	8CR0.	Asthma clin management plan
21262	Asthma resolved	8H2P.	Emergency admission, asthma
14B4.	H/O: asthma	8HTT.	Referral to asthma clinic
14Ok0	At risk sevre asthma exacrbatn	9hA..	Except report: asthma qual ind
173A.	Exercise induced asthma	9hA1.	Except asthma qual ind: Pt uns
173c.	Occupational asthma	9hA2.	Excep asthma qual ind: Inf dis
173d.	Work aggravated asthma	9N1d.	Seen in asthma clinic
178..	Asthma trigger	9N1d0	Seen in school asthma clinic
178A.	Asthma trigger - airborne dust	9N4Q.	Did not attend asthma clinic
178B.	Asthma trigger - exercise	9NI8.	Asthma outreach clinic
1O2..	Asthma confirmed	9NNX.	Under care asthma spclst nurse
212G.	Asthma resolved	9OJ..	Asthma monitoring admin.
388t.	RCP asthma assessment	9OJ1.	Attends asthma monitoring
38B8.	Sevr asthma exacer risk assess	9OJ2.	Refuses asthma monitoring
38DL.	Asthma control test	9OJ3.	Asthma monitor offer default
38DT.	Asthma control questionnaire	9OJ4.	Asthma monitor 1st letter
38DV.	Mini asthma QOL questionnaire	9OJ5.	Asthma monitor 2nd letter
38QM.	Childhood Asthma Control Test	9OJ6.	Asthma monitor 3rd letter
633y.	Num asthm exacer in past year	9OJ7.	Asthma monitor verbal invite
661M1	Asthma self-manage plan agreed	9OJ8.	Asthma monitor phone invite
661N1	Asthma self-manage plan review	9OJ9.	Asthma monitoring deleted

Evans A, *et al. Thorax* 2021; 76:116–125. doi: 10.1136/thoraxjnl-2020-215422

Table S3: General Practice diagnosis codes for Asthma and Wheeze (cont)

**General Practice Read codes v2 for Asthma Diagnosis and Wheeze symptoms (includes any diagnosis, symptom or procedure codes, extracted 2017)**			
**Asthma diagnosis**			
**Code**	**Description**	**Code**	**Description**
663e.	Asthma restricts exercise	9OJA.	Asthma monitoring check done
663e1	Asthma severely restr exercise	9OJB.	Asthma monitr invt SMS txt msg
663f.	Asthma never restrcts exercise	9OJB0	Asthma monitrng SMS 1st invit
663j.	Asthma - currently active	9OJB1	Asthma monitrng SMS 2nd invit
663N.	Asthma disturbing sleep	9OJB2	Asthma monitrng SMS 3rd invit
663N0	Asthma causing night waking	9OJC.	Asthma monitrng invitatn email
663N1	Asthma disturbs sleep weekly	9OJZ.	Asthma monitoring admin.NOS
663N2	Asthma disturbs sleep freqntly	9Q21.	Patient in asthma study
663O.	Asthma not disturbing sleep	TJF7.	AR - antiasthmatics
663O0	Asthma never disturbs sleep	TJF73	AR - theophylline (asthma)
663P.	Asthma limiting activities	TJF7z	AR - antiasthmatic NOS
663P0	Asthma limit act 1-2 time mth		
**Wheeze diagnosis**			
**Code**	**Description**		
1737.	Wheezing		
17370	Constant wheezing		
17371	Wheezing in absence of colds		
173B.	Nocturnal cough / wheeze		
173e.	Viral wheeze		
2326.	O/E - expiratory wheeze		
232H.	O/E inspiratory wheeze		
6635.	Increasing exercise wheeze		
R0609	Wheezing		
R060E	Mild wheeze		
R060F	Moderate wheeze		
R060G	Severe wheeze		
R060H	Very severe wheeze		

Table S4: General Practice prescription codes for Asthma

General Practice Read Code Version 2 Asthma prescriptions (list from the Asthma in Swansea study 2009[24]) that have been categorised into diagnosis only, intermittent bronchodilator, persistent mild, persistent moderate and persistent severe prescription types (update extracted 2017).

Mild asthma: bronchodilators

Drug Category	Description
1	Short-acting beta$_2$ agonist and short-acting muscarinic antagonist
8	Xanthine bronchodilator assumed to be short-acting (not specified as injectable or with modified release^).
9	Bronchodilator + decongestant
15	Short-acting xanthine bronchodilator + Epinephrine, or Adrenaline inhaler
16	Theophylline, Ephedrine & Phenobarbital combination (relievers)
17	Nebuliser of reliever medication (code 1 in other forms)*

Severe Asthma: Injections and immunosuppressants or similar

Drug Category	Description
5	Injection prescription (patient probably hospitalised for treatment with injection)
21	Omalizumab and brand names, immunosuppressant IgE: mainly for allergic-asthma but can be for spontaneous hives (not in Cost of Asthma study coding).

*young children may need medication to be administered with a nebuliser as inhalers may be difficult to use e.g. for an asthma attack; older children and adults would usually only receive nebuliser treatment in hospital.
^ medication with modified release has a delayed release.

Moderate asthma medications: Inhaled corticosteroids, long-acting beta$_2$ agonists, leukotrienes or alternatives (excluding Asthma-related antihistamines coded as 12)

Drug Category	Description
2	Inhaled corticosteroid (ICS)
3	Long acting beta$_2$ agonist
4	Leukotriene receptor antagonist
6	Long-acting beta$_2$ agonist + corticosteroid
7	Long-acting antimuscarinic (also known as antichloinergic)
10	Short-acting beta$_2$ agonist + corticosteroid
11	Short-acting beta$_2$ agonist + non-steroid alternative
13	Non-steroid alternative
14	Other long-acting bronchodilator
18	Nebuliser of preventer medication (code 3 in other forms)*
19	Nebuliser ICS (code 2 other forms)*
20	Nebuliser non-steroid alternative (code as 13 in other forms)*
26	Long-acting xanthine bronchodilator (modified release^)

Read Code v2	Description	Drug category	Read Code v2	Description	Drug category
c1	SELECTIVE BETA-ADRENOCEPTOR STIMULANT	1	c43a	SLO-PHYLLIN 250mg m/r capsules	26
c11	SALBUTAMOL [ORAL PREPARATIONS]	1	c43A	THEOPHYLLINE 200mg/10mL injection	5
c111	*ASMAVEN 2mg tablets	1	c43b	*THEO-DUR 200mg m/r tablets	26
c112	*ASMAVEN 4mg tablets	1	c43B	THEOPHYLLINE 10mg/5mL sugar free solution	8
c113	*COBUTOLIN 2mg tablets	1	c43c	*THEO-DUR 300mg m/r tablets	26
c114	*COBUTOLIN 4mg tablets	1	c43d	*THEOGRAD 350mg m/r tablets	26
c115	*SALBULIN 2mg tablets	1	c43e	UNIPHYLLIN CONTINUS 400mg m/r tablets	26
c116	*SALBULIN 4mg tablets	1	c43f	UNIPHYLLIN CONTINUS 200mg m/r tablets	26
c117	*SALBULIN 2mg/2mL liquid	1	c43g	LABOPHYLLINE 200mg/10mL injection	5
c118	*VENTOLIN 2mg tablets	1	c43h	UNIPHYLLIN CONTINUS 300mg m/r tablets	26
c119	*VENTOLIN 4mg tablets	1	c43i	*BIOPHYLLINE 350mg m/r tablets	26

Evans A, *et al. Thorax* 2021; 76:116–125. doi: 10.1136/thoraxjnl-2020-215422

Table S4: General Practice prescription codes for Asthma (cont)

General Practice Read Code Version 2 Asthma prescriptions (list from the Asthma in Swansea study 2009[24]) that have been categorised into diagnosis only, intermittent bronchodilator, persistent mild, persistent moderate and persistent severe prescription types (update extracted 2017).

Read Code v2	Description	Drug category	Read Code v2	Description	Drug category
c11a	*VENTOLIN 8mg m/r tablets	1	c43j	*BIOPHYLLINE 500mg m/r tablets	26
c11A	*VENTOLIN CR 4mg m/r tablets	1	c43k	THEOPHYLLINE 500mg m/r tablets	26
c11B	*SALBUTAMOL 4mg m/r tablets	1	c43m	*THEOPHYLLINE 125mg/5mL syrup	8
c11b	VENTOLIN 2mg/5mL syrup	1	c43n	*THEOPHYLLINE 125mg tablets	8
c11C	*VENTOLIN CR 8mg m/r tablets	1	c43o	*THEOPHYLLINE 60mg/5mL liquid	8
c11c	VOLMAX 4mg m/r tablets	1	c43p	THEOPHYLLINE 175mg m/r tablets	26
c11D	SALAPIN 2mg/5mL sugar free syrup	1	c43q	THEOPHYLLINE 250mg m/r tablets	26
c11d	VOLMAX 8mg m/r tablets	1	c43r	THEOPHYLLINE 300mg m/r capsules	26
c11e	*SALBUVENT 2mg tablets	1	c43s	THEOPHYLLINE 60mg m/r capsules	26
c11f	*SALBUVENT 4mg tablets	1	c43t	THEOPHYLLINE 125mg m/r capsules	26
c11g	*SALBUVENT 2mg/5mL syrup	1	c43u	THEOPHYLLINE 250mg m/r capsules	26
c11h	SALBUVENT 2mg/5mL syrup 2litre	1	c43v	THEOPHYLLINE 200mg m/r tablets	26
c11i	*VENTOLIN CR 4mg m/r tablets	1	c43w	THEOPHYLLINE 300mg m/r tablets	26
c11j	SALBUTAMOL 4mg m/r tablets	1	c43x	THEOPHYLLINE 350mg m/r tablets	26
c11k	*VENTOLIN CR 8mg m/r tablets	1	c43y	THEOPHYLLINE 400mg m/r tablets	26
c11m	LIBETIST 2mg/5mL sugar free syrup	1	c43z	*THEOPHYLLINE 200mg tablets	8
c11n	SALBUTAMOL 4mg m/r capsules	1	c44	CAFFEINE	8
c11o	SALBUTAMOL 8mg m/r capsules	1	c441	CAFFEINE solution	8
c11p	VENTMAX SR 4mg m/r capsules	1	c442	CAFFEINE CITRATE solution	8
c11q	VENTMAX SR 8mg m/r capsules	1	c5	COMPOUND BRONCHODILATORS	1
c11v	SALBUTAMOL 4mg tablets	1	c51	COMPOUND BRONCHODILATORS A-Z	1
c11w	*SALBUTAMOL 2mg/2mL liquid	1	c511	ADRENALINE+ATROPINE COMPOUND spray	17
c11x	SALBUTAMOL 2mg tablets	1	c512	ALUPENT EXPECTORANT 20mg tablets	1
c11y	SALBUTAMOL 8mg m/r tablets	1	c513	*ALUPENT EXPECTORANT mixture	1
c11z	SALBUTAMOL 2mg/5mL sugar free syrup	1	c514	*ASMA-VYDRIN spray	15
c12	SALBUTAMOL [PARENTERAL PREPARATIONS]	1	c515	*ASMA-VYDRIN spray 120mL	15
c121	VENTOLIN 250micrograms/5mL injection	5	c516	*BRICANYL COMPOUND tablets	1
c122	VENTOLIN 500microgram/1mL injection	5	c517	*BRICANYL EXPECTORANT elixir	1
c123	VENTOLIN 5mg/5mL intravenous infusion	5	c518	*BRONCHILATOR inhaler	1
c124	SALBUVENT 250microgram/5mL injection	5	c519	*BROVON spray 20mL	15
c125	SALBUVENT 500micrograms/1mL injection	5	c51a	*BROVON spray 50mL	15
c126	SALBUVENT 5mg/5mL intravenous infusion	5	c51A	FENOTEROL+IPRATROPIUM 100micrograms/40micrograms inhaler	1
c12w	*SALBUTAMOL 5mg/50mL injection	5	c51b	*BROVON MIDGET inhaler	15
c12x	SALBUTAMOL 250micrograms/5mL injection	5	c51B	FENOTEROL+IPRATROPIUM 100mcg/40mcg breath-act aerosl inhaler	1
c12y	SALBUTAMOL 500microgram/1mL injection	5	c51c	BROVON RESERVOIR+CLOSURE	15
c12z	SALBUTAMOL 5mg/5mL intravenous infusion	5	c51C	SALBUTAMOL+IPRATROPIUM 100micrograms/20micrograms	1
c13	SALBUTAMOL [INHALATION PREPRATIONS]	1	c51d	BROVON RUBBER BULB	15
c131	*ASMAVEN 100micrograms inhaler	1	c51D	COMBIVENT inhaler	1

Evans A, *et al. Thorax* 2021; 76:116–125. doi: 10.1136/thoraxjnl-2020-215422

Table S4: General Practice prescription codes for Asthma (cont)

General Practice Read Code Version 2 Asthma prescriptions (list from the Asthma in Swansea study 2009[24]) that have been categorised into diagnosis only, intermittent bronchodilator, persistent mild, persistent moderate and persistent severe prescription types (update extracted 2017).

Read Code v2	Description	Drug category	Read Code v2	Description	Drug category
c132	COBUTOLIN 100microgram inhaler	1	c51e	*BROVON pressurised inhaler	15
c133	SALBULIN 100micrograms inhaler	1	c51E	COMBIVENT Unit Dose Vials	1
c134	VENTOLIN 100micrograms inhaler	1	c51f	*CAM mixture	1
c135	VENTOLIN 2.5mg/2.5mL Nebules	17	c51F	SALBUTAMOL+IPRATROPIUM 2.5mg/500mcg nebulisation units	17
c136	VENTOLIN 200micrograms rotacaps	1	c51g	*DUO-AUTOHALER inhaler	9
c137	VENTOLIN 400micrograms rotacaps	1	c51h	DUO-AUTOHALER refill cannister	9
c138	ROTAHALER DEVICE	1	c51H	IPRATROPIUM BROMIDE+SALBUTAMOL 500mcg/2.5mg nebuliser	17
c139	VENTOLIN 100mg/20mL respirator solution	17	c51i	*DUOVENT inhaler	1
c13a	AEROLIN-400 100microgram inhaler	1	c51j	ISO-BROVON pressurised inhaler	15
c13A	STERI-NEB SALAMOL 2.5mg nebulisation units	17	c51k	ISO-BROVON PLUS pressurised inhaler	15
c13b	*ROTAHALER DEVICE	1	c51l	*MEDIHALER DUO inhaler	9
c13B	STERI-NEB SALAMOL 5mg nebulisation units	17	c51m	*NETHAPRIN DOSPAN m/r tablets	14
c13c	AEROLIN AUTO 100microgram inhaler	1	c51n	RYBARVIN INHALANT solution 30mL	15
c13C	SALBUTAMOL 200micrograms disks+disk inhaler	1	c51o	*RYBAR NO-1 inhaler	12
c13D	SALBUTAMOL 400micrograms disks+disk inhaler	1	c51p	*RYBAR NO-2 inhaler	12
c13d	VENTODISKS 200micrograms diskhaler 14x8	1	c51q	*TAUMASTHMAN tablets	16
c13E	SALBUTAMOL 200micrograms disk refill	1	c51r	*TEDRAL tablets	16
c13e	VENTODISKS 400micrograms diskhaler 14x8	1	c51s	*TEDRAL elixir	16
c13F	SALBUTAMOL 400micrograms disk refill	1	c51t	*FRANOL NEW 11mg/120mg tablets	15
c13f	VENTODISKS 200micrograms disk refill 14x8	1	c51u	FRANOL PLUS NEW 15mg/120mg tablets	15
c13G	SALBUTAMOL 100micrograms breath-act aerosol inhaler	1	c51v	DUOVENT UDV nebuliser solution 4mL	17
c13g	VENTODISKS 400micrograms disk refill 14x8	1	c51w	IPRATROPIUM BR+FENOTEROL HBR 500mcg/1.25mg neb solution	17
c13H	*SALAMOL 100micrograms inhaler	1	c51x	*DUOVENT Autohaler	1
c13h	SALBUVENT 100micrograms inhaler	1	c52	*BRONCHODILATORS + SEDATIVE	1
c13I	AIROMIR 100micrograms CFC-free inhaler	1	c521	*FRANOL OLD tablets	15
c13i	SALBUVENT RONDO 100microgram inhaler	1	c522	*FRANOL PLUS OLD tablets	15
c13J	SALBUTAMOL 100micrograms CFC-free inhaler	1	c523	*FRANOL EXPECTORANT elixir	15
c13j	SALBUVENT 5mg/mL respirator solution	17	c53	COMPOUND BRONCHODILATORS [1]	2
c13k	*SALBUVENT RONDO spacer x1	1	c531	IPRAMOL STERI-NEB 2.5mg/500micrograms nebuliser soln 2.5mL	17
c13K	SALAMOL EASI-BREATHE 100mcg breath-actuated aerosol inhaler	1	c6	CORTICOSTEROIDS [RESPIRATORY USE]	2
c13l	AEROLIN 100micrograms Autohaler 200d	1	c61	BECLOMETASONE DIPROPIONATE [RESPIRATORY USE]	2
c13L	SALBUTAMOL 200micrograms breath-actuated dry powder inhaler	1	c611	BECLOFORTE 250microgram inhaler	2
c13M	VENTOLIN 200micrograms Accuhaler	1	c612	BECOTIDE-50 50microgram inhaler	2
c13m	VENTOLIN 5mg/2.5mL Nebules	17	c613	BECOTIDE 100micrograms rotacaps	2
c13n	AEROLIN 100micrograms Autohaler 100d	1	c614	BECOTIDE 200micrograms rotacaps	2
c13N	SALBUTAMOL 100micrograms vortex metered dose inhaler	1	c615	*BECOTIDE rotahaler device	2
c13o	SALBUTAMOL 5mg/2.5mL nebulisation units	17	c616	BECOTIDE 50micrograms/mL nebuliser solution	19
c13O	VENTOLIN EASI-BREATHE 100microgram inhaler	1	c617	BECOTIDE-100 100microgram inhaler	2

Evans A, *et al. Thorax* 2021; 76:116–125. doi: 10.1136/thoraxjnl-2020-215422

Table S4: General Practice prescription codes for Asthma (cont)

General Practice Read Code Version 2 Asthma prescriptions (list from the Asthma in Swansea study 2009[24]) that have been categorised into diagnosis only, intermittent bronchodilator, persistent mild, persistent moderate and persistent severe prescription types (update extracted 2017).

Read Code v2	Description	Drug category	Read Code v2	Description	Drug category
c13p	MAXIVENT 100microgram inhaler	1	c618	*VOLUMATIC spacer device	1
c13P	SALBUTAMOL 100micrograms Spacehaler	1	c619	BECODISK 100micrograms diskhaler 14x8	2
c13Q	ASMASAL 95micrograms Clickhaler	1	c61A	BECLOMETASONE DIPROPIONATE 400micrograms disks+disk	2
c13q	SALBUTAMOL 200 Cyclocaps	1	c61a	BECODISK 200micrograms diskhaler 14x8	2
c13R	SALBUTAMOL 100micrograms breath-act dry powder inhaler	1	c61B	BECLOMETASONE DIPROPIONATE 400micrograms disk refill	2
c13r	SALBUTAMOL 400 Cyclocaps	1	c61b	BECOTIDE 400micrograms rotacaps	2
c13s	*VENTOLIN rotahaler device	1	c61C	BECLOMETHASONE DIPROPIONATE 250mcg inhaler+spacer device	2
c13S	SALBUTAMOL 95micrograms breath-actuated dry powder inhaler	1	c61c	BECODISK 100micrograms disk refill 14x8	2
c13T	VENTOLIN 100micrograms Evohaler	1	c61D	BECLOMETASONE DIPROP 50mcg breath-actuated aerosol inhaler	2
c13U	SALBUTAMOL 100mcg CFC-free breath-actuated aerosol inhaler	1	c61d	BECODISK 200micrograms disk refill 14x8	2
c13V	AIROMIR 100micrograms CFC-free Autohaler	1	c61E	BECLOMETASONE DIPROP 250mcg breath-actuated aerosol inhaler	2
c13v	SALBUTAMOL 100microgram inhaler	1	c61e	BECODISK 400micrograms diskhaler 7x8	2
c13W	MAXIVENT 2.5mg/2.5mL Steripoules	17	c61F	BECLOMETASONE DIPROP 100mcg breath-actuated aerosol inhaler	2
c13w	SALBUTAMOL 2.5mg/2.5mL nebulisation units	17	c61f	BECODISK 400micrograms disk refill 7x8	2
c13X	MAXIVENT 5mg/2.5mL Steripoules	17	c61g	BECLOFORTE VM 250micrograms inhaler+volumatic	2
c13x	SALBUTAMOL 200micrograms inhalation capsules	1	c61G	FILAIR 50micrograms inhaler	2
c13Y	SALBULIN 100micrograms CFC-free inhaler	1	c61h	BECLOMETASONE DIPROPIONATE 400micrograms inhalation	2
c13y	SALBUTAMOL 400micrograms inhalation capsules	1	c61H	FILAIR 100micrograms inhaler	2
c13Z	SALAMOL 100micrograms CFC-free inhaler	1	c61i	BECOTIDE-200 200microgram inhaler	2
c13z	SALBUTAMOL 100mg/20mL respirator solution	17	c61j	AEROBEC 50microgram Autohaler	2
c14	TERBUTALINE SULPHATE [RESPIRATORY USE]	1	c61J	FILAIR FORTE 250micrograms inhaler	2
c141	BRICANYL 5mg tablets	1	c61k	AEROBEC FORTE 250microgram Autohaler	2
c142	BRICANYL 1.5mg/5mL syrup	1	c61K	BECLAZONE 50micrograms inhaler	2
c143	BRICANYL 500micrograms/1mL injection	5	c61l	AEROBEC 100microgram Autohaler	2
c144	BRICANYL 250micrograms inhaler	1	c61L	BECLAZONE 100micrograms inhaler	2
c145	BRICANYL 250micrograms refill cannister	1	c61M	BECLAZONE 250micrograms inhaler	2
c146	BRICANYL 250micrograms spacer inhaler	1	c61m	BECLOFORTE DISKHALER 400micrograms 14x8	2
c147	BRICANYL RESPULES 5mg/2mL nebuliser solution	17	c61N	BECLAZONE 50 EASI-BREATHE inhaler	2
c148	BRICANYL 100mg/10mL respirator solution	17	c61n	BECLOFORTE DISKS 400micrograms disk refill 14x8	2
c149	*BRICANYL SA 7.5mg m/r tablets	1	c61O	BECLAZONE 100 EASI-BREATHE inhaler	2
c14a	*MONOVENT 5mg tablets	1	c61P	BECLAZONE 250 EASI-BREATHE inhaler	2
c14b	*MONOVENT 1.5mg/5mL syrup	1	c61p	BECLOMETASONE DIPROPIONATE 100micrograms disks+disk	2
c14c	*MONOVENT SA 7.5mg m/r tablets	1	c61Q	BECLOFORTE INTEGRA 250micrograms inhaler+compact spacer	2
c14d	*NEBUHALER spacer device	1	c61q	BECLOMETASONE DIPROPIONATE 200micrograms disks+disk	2
c14e	BRICANYL 2.5mg/5mL injection	5	c61R	BECLOFORTE INTEGRA 250micrograms refill	2
c14f	BRICANYL 500micrograms Turbohaler	1	c61r	BECLOMETASONE DIPROPIONATE 100micrograms disk refill	2
c14g	TERBUTALINE 500micrograms inhaler	1	c61s	BECLOMETASONE DIPROPIONATE 200micrograms disk refill	2
c14h	TERBUTALINE 2.5mg/5mL injection	5	c61S	BECLOMETHASONE DIPROPIONATE 250mcg inhaler+compact	2

Evans A, *et al. Thorax* 2021; 76:116–125. doi: 10.1136/thoraxjnl-2020-215422

Table S4: General Practice prescription codes for Asthma (cont)

General Practice Read Code Version 2 Asthma prescriptions (list from the Asthma in Swansea study 2009[24]) that have been categorised into diagnosis only, intermittent bronchodilator, persistent mild, persistent moderate and persistent severe prescription types (update extracted 2017).

Read Code v2	Description	Drug category	Read Code v2	Description	Drug category
c14i	TERBUTALINE SULPHATE 200mg/20mL nebuliser solution	17	c61t	BECLOMETASONE DIPROPIONATE 250micrograms inhaler	2
c14j	TERBUTALINE 500micrograms breath-actuated dry powder inhaler	1	c61T	BECLOMETHASONE DIPROPIONATE 250mcg compact spacer refill	2
c14k	BRICANYL 200mg/20mL respirator solution	17	c61u	BECLOMETASONE DIPROPIONATE 200micrograms inhaler	2
c14r	TERBUTALINE 5mg tablets	1	c61U	BECLOMETHASONE rotahaler device	2
c14s	TERBUTALINE 500microgram/1mL injection	5	c61v	BECLOMETASONE DIPROPIONATE 50micrograms inhaler	2
c14t	TERBUTALINE 250micrograms inhaler	1	c61V	BECLOMETHASONE DIPROPIONATE 50mcg vortex metered dose	2
c14u	TERBUTALINE 250micrograms refill cannister	17	c61W	*BDP 50micrograms Spacehaler	2
c14v	TERBUTALINE 250micrograms spacer	1	c61w	BECLOMETASONE DIPROPIONATE 100micrograms inhalation	2
c14w	TERBUTALINE 5mg/2mL nebuliser solution	17	c61x	BECLOMETASONE DIPROPIONATE 200micrograms inhalation	2
c14x	TERBUTALINE 100mg/10mL respirator solution	17	c61X	BECLOMETHASONE DIPROPIONATE 100mcg vortex metered dose	2
c14y	*TERBUTALINE 7.5mg m/r tablets	1	c61Y	*BDP 100micrograms Spacehaler	2
c14z	TERBUTALINE 1.5mg/5mL syrup	1	c61y	BECLOMETHASONE DIPROPIONATE 50mcg/mL nebuliser solution	19
c15	FENOTEROL HYDROBROMIDE	1	c61z	BECLOMETASONE DIPROPIONATE 100micrograms inhaler	2
c151	*BEROTEC 200micrograms inhaler	1	c61Z	BECLOMETHASONE DIPROPIONATE 250mcg vortex metered dose	2
c152	BEROTEC 100mg/20mL respirator solution	17	c62	BECLOMETASONE COMPOUNDS	2
c153	*BEROTEC 100micrograms inhaler	1	c621	*VENTIDE inhaler	10
c154	FENOTEROL 100micrograms inhaler	1	c622	*VENTIDE Rotacaps	10
c15y	FENOTEROL 200micrograms inhaler	1	c623	*VENTIDE paediatric Rotacaps	10
c15z	FENOTEROL 100mg/20mL respirator solution	17	c624	*VENTIDE Rotahaler device	10
c16	PIRBUTEROL	1	c63	*BETAMETHASONE VALERATE	2
c161	*EXIREL 10mg capsules	1	c631	*BEXTASOL 100microgram inhaler	2
c162	*EXIREL 15mg capsules	1	c63z	BETAMETHASONE 100micrograms inhaler	2
c163	*EXIREL 7.5mg/5mL syrup	1	c64	BUDESONIDE [RESPIRATORY USE]	2
c164	*EXIREL 200micrograms inhaler	1	c641	PULMICORT 200microgram inhaler 200dose	2
c16w	*PIRBUTEROL 10mg capsules	1	c642	PULMICORT 200micrograms refill 100dose	2
c16x	*PIRBUTEROL 15mg capsules	1	c643	PULMICORT 200micrograms refill 200dose	2
c16y	*PIRBUTEROL 7.5mg/5mL syrup	1	c644	PULMICORT LS 50microgram inhaler	2
c16z	PIRBUTEROL 200micrograms inhaler	1	c645	PULMICORT LS 50micrograms refill	2
c17	REPROTEROL HYDROCHLORIDE	3	c646	*NEBUHALER spacer device	1
c171	*BRONCHODIL 20mg tablets	3	c647	PULMICORT 200microgram inhaler 100dose	2
c172	*BRONCHODIL 10mg/5mL elixir	3	c648	PULMICORT 200microgram Turbohaler 100dose	2
c173	BRONCHODIL 500micrograms inhaler	3	c649	PULMICORT 400microgram Turbohaler 50dose	2
c174	BRONCHODIL 10mg/mL respirator solution	3	c64A	BUDESONIDE 200micrograms refill cannister	2
c17w	*REPROTEROL 20mg tablets	3	c64a	PULMICORT 500micrograms Respules 2mL unit	2
c17x	*REPROTEROL 10mg/5mL elixir	3	c64B	BUDESONIDE 50micrograms spacer inhaler	2
c17y	REPROTEROL 500micrograms inhaler	3	c64b	PULMICORT 1mg Respules 2mL unit	2
c17z	REPROTEROL 10mg/mL respirator solution	18	c64c	PULMICORT 100microgram Turbohaler 200dose	2
c18	RIMITEROL HYDROBROMIDE	1	c64C	PULMICORT 200micrograms spacer inhaler	2
c181	PULMADIL 200micrograms inhaler	1	c64d	BUDESONIDE 100micrograms breath-actuated dry powder inhaler	2

Table S4: General Practice prescription codes for Asthma (cont)

General Practice Read Code Version 2 Asthma prescriptions (list from the Asthma in Swansea study 2009[24]) that have been categorised into diagnosis only, intermittent bronchodilator, persistent mild, persistent moderate and persistent severe prescription types (update extracted 2017).

Read Code v2	Description	Drug category	Read Code v2	Description	Drug category
c182	PULMADIL 200micrograms autohaler	1	c64D	PULMICORT LS 50micrograms spacer inhaler	2
c183	PULMADIL 200micrograms auto refill cannister	1	c64e	BUDESONIDE 50micrograms refill cannister	2
c184	RIMITEROL 200micrograms breath-actuated aerosol inhaler	1	c64E	PULMICORT 200micrograms inhaler with NebuChamber	2
c18y	RIMITEROL 200micrograms inhaler	1	c64F	BUDESONIDE 200micrograms/dose dry powder cartridge refill	2
c18z	RIMITEROL 200micrograms auto refill cannister	1	c64g	BUDESONIDE 200micrograms breath-actuated dry powder inhaler	2
c19	SALMETEROL XINAFOATE	3	c64G	NOVOLIZER BUDESONIDE 200micrograms/dose cartridge refill	2
c191	SALMETEROL 25microgram inhaler	3	c64h	BUDESONIDE 400micrograms breath-actuated dry powder inhaler	2
c192	*SEREVENT 25microgram inhaler	3	c64H	EASYHALER BUDESONIDE 100mcg breath-actuated dry powder inh	2
c193	SEREVENT 50microgram diskhaler	3	c64i	BUDESONIDE 500micrograms/2mL nebuliser solution	19
c194	SEREVENT 50micrograms disk refill	3	c64I	EASYHALER BUDESONIDE 200mcg breath-actuated dry powder inh	2
c195	SALMETEROL 50micrograms disks+disk inhaler	3	c64j	BUDESONIDE 1mg/2mL nebuliser solution	19
c196	SALMETEROL 50micrograms disk refill	3	c64J	EASYHALER BUDESONIDE 400mcg breath-actuated dry powder inh	2
c197	SALMETEROL 50micrograms breath-actuated dry powder inhaler	3	c64k	BUDESONIDE 200 Cyclocaps	2
c198	SEREVENT 50micrograms Accuhaler	3	c64K	PULMICORT 100micrograms CFC-free inhaler	2
c199	SEREVENT 25micrograms Evohaler	3	c64L	BUDESONIDE 100micrograms CFC-free inhaler	2
c19A	NEOVENT 25micrograms CFC-free inhaler	3	c64l	BUDESONIDE 400 Cyclocaps	2
c19B	VERTINE 25micrograms CFC-free inhaler	3	c64m	BUDESONIDE 200micrograms inhalation capsules	2
c19z	SALMETEROL 25micrograms CFC-free inhaler	3	c64M	PULMICORT 200micrograms CFC-free inhaler	2
c1A	SALINE FOR NEBULISATION	1	c64N	BUDESONIDE 200micrograms CFC-free inhaler	2
c1a	TULOBUTEROL HYDROCHLORIDE	3	c64n	BUDESONIDE 400micrograms inhalation capsules	2
c1a1	*TULOBUTEROL 2mg tablets	3	c64o	BUDESONIDE 200micrograms inhaler with spacer device	2
c1A1	STERI-NEB SALINE 0.9% nebules	1	c64p	NOVOLIZER BUDESONIDE 200micrograms/dose cartridge+inhaler	2
c1a2	*BRELOMAX 2mg tablets	3	c64u	BUDESONIDE 200micrograms/dose dry powder cartridge+inhaler	2
c1A2	SODIUM CHLORIDE 0.9% nebules	1	c64v	BUDESONIDE 200microgram inhaler	2
c1a3	*RESPACAL 2mg tablets	3	c64w	*BUDESONIDE refill 100dose	2
c1A3	SODIUM CHLORIDE 6% solution for inhalation	1	c64x	*BUDESONIDE refill 200dose	2
c1a4	TULOBUTEROL 1mg/5mL sugar free liquid	3	c64y	BUDESONIDE 50microgram inhaler	2
c1a5	RESPACAL 1mg/5mL sugar free liquid	3	c64z	BUDESONIDE 200micrograms spacer inhaler	2
c1A5	SODIUM CHLORIDE 0.9% Steripoules	1	c65	FLUTICASONE PROPIONATE [RESPIRATORY USE]	2
c1A6	SALINE 0.9% Steripoules 2.5ml	1	c651	FLIXOTIDE 50micrograms diskhaler	2
c1A7	SODIUM CHLORIDE 7% nebuliser solution	1	c652	FLIXOTIDE 100micrograms diskhaler	2
c1A8	NEBUSAL 7% hypertonic sodium chloride nebuliser solution	1	c653	FLIXOTIDE 250micrograms diskhaler	2
c1AA	SODIUM CHLORIDE 3% solution for inhalation	1	c654	FLUTICASONE PROPIONATE 50micrograms disks+disk inhaler	2
c1B	BAMBUTEROL HYDROCHLORIDE	3	c655	FLUTICASONE PROPIONATE 100micrograms disks+disk inhaler	2
c1B1	BAMBEC 10mg tablets	3	c656	FLUTICASONE PROPIONATE 250micrograms disks+disk inhaler	2
c1B2	BAMBEC 20mg tablets	3	c657	FLIXOTIDE 50micrograms disk refill	2
c1B3	BAMBUTEROL HYDROCHLORIDE 10mg tablets	3	c658	FLIXOTIDE 100micrograms disk refill	2
c1B4	BAMBUTEROL HYDROCHLORIDE 20mg tablets	3	c659	FLIXOTIDE 250micrograms disk refill	2
c1c	FLUTICASONE PROPIONATE+FORMOTEROL FUMARATE	6	c65a	FLIXOTIDE 2mg/2mL Nebules	19

Evans A, *et al. Thorax* 2021; 76:116–125. doi: 10.1136/thoraxjnl-2020-215422

Table S4: General Practice prescription codes for Asthma (cont)

General Practice Read Code Version 2 Asthma prescriptions (list from the Asthma in Swansea study 2009[24]) that have been categorised into diagnosis only, intermittent bronchodilator, persistent mild, persistent moderate and persistent severe prescription types (update extracted 2017).

Read Code v2	Description	Drug category	Read Code v2	Description	Drug category
c1C	FORMOTEROL	3	c65A	FLUTICASONE PROPIONATE 50micrograms disk refill	2
c1c1	FLUTIFORM 50micrograms/5micrograms inhaler	2	c65B	FLUTICASONE PROPIONATE 100micrograms disk refill	2
c1C1	FORMOTEROL FUMARATE 12micrograms inhalation	3	c65b	FLUTICASONE PROPIONATE 125micrograms CFC-free inhaler	2
c1c2	FLUTIFORM 125micrograms/5micrograms inhaler	2	c65c	FLUTICASONE PROPIONATE 250micrograms CFC-free inhaler	2
c1C2	FORADIL 12micrograms inhalation capsules+inhaler	3	c65C	FLUTICASONE PROPIONATE 250micrograms disk refill	2
c1c3	FLUTIFORM 250micrograms/10micrograms inhaler	2	c65d	FLIXOTIDE 125micrograms Evohaler	2
c1C3	FORMOTEROL FUMARATE 6micrograms breath-act dry powder inh	3	c65D	FLIXOTIDE 25micrograms inhaler	2
c1C4	FORMOTEROL FUMARATE 12micrograms breath-act dry powder inh	3	c65e	FLIXOTIDE 250micrograms Evohaler	2
c1C5	OXIS 6micrograms Turbohaler	3	c65E	FLIXOTIDE 50micrograms inhaler	2
c1C6	OXIS 12micrograms Turbohaler	3	c65F	FLIXOTIDE 125micrograms inhaler	2
c1C7	ATIMOS MODULITE 12micrograms metered dose inhaler	3	c65f	FLUTICASONE PROPIONATE 50micrograms CFC-free inhaler	2
c1C8	FORMOTEROL EASYHALER 12micrograms breath-act dry powder	3	c65g	FLIXOTIDE 50micrograms Evohaler	2
c1cx	FLUTICASONE PROPIONATE+FORMOTEROL FUMARATE	6	c65G	FLUTICASONE PROPIONATE 25micrograms inhaler	2
c1cy	FLUTICASONE PROPIONATE+FORMOTEROL FUMARATE	6	c65H	FLUTICASONE PROPIONATE 50micrograms inhaler	2
c1Cy	FORMOTEROL FUMARATE DIHYDRATE 12mcg breath-act dry pdr	3	c65I	FLUTICASONE PROPIONATE 125micrograms inhaler	2
c1cz	FLUTICASONE PROPIONATE+FORMOTEROL FUMARATE	6	c65J	FLUTICASONE PROPIONATE 250micrograms inhaler	2
c1Cz	FORMOTEROL FUMARATE DIHYDRATE 12mcg metered dose	3	c65K	FLIXOTIDE 250micrograms inhaler	2
c1D	SALMETEROL+FLUTICASONE PROPIONATE	6	c65L	FLIXOTIDE 500micrograms diskhaler	2
c1D1	SERETIDE 100 Accuhaler	6	c65M	FLIXOTIDE 500micrograms disk refill	2
c1D2	SERETIDE 250 Accuhaler	6	c65N	FLUTICASONE PROPIONATE 500micrograms disks+disk inhaler	2
c1D3	SERETIDE 500 Accuhaler	6	c65O	FLUTICASONE PROPIONATE 500micrograms disk refill	2
c1D4	SERETIDE 50 Evohaler	6	c65P	FLUTICASONE PROPIONATE 50mcg breath-actuated dry powder inh	2
c1D5	SERETIDE 125 Evohaler	6	c65Q	FLUTICASONE PROPIONATE 100mcg breath-actuated dry powder inh	2
c1D6	SERETIDE 250 Evohaler	6	c65R	FLUTICASONE PROPIONATE 250mcg breath-actuated dry powder inh	2
c1D7	SIRDUPLA 25micrograms/125micrograms inhaler	6	c65S	FLUTICASONE PROPIONATE 500mcg breath-actuated dry powder inh	2
c1D8	SIRDUPLA 25micrograms/250micrograms inhaler	6	c65T	FLIXOTIDE 50micrograms Accuhaler	2
c1Du	SALMETEROL+FLUTICASONE PROPIONATE 25mcg/50mcg CFC-	6	c65U	FLIXOTIDE 100micrograms Accuhaler	2
c1Dv	SALMETEROL+FLUTICASONE PROPIONATE 25mcg/125mcg CFC-	6	c65V	FLIXOTIDE 250micrograms Accuhaler	2
c1Dw	SALMETEROL+FLUTICASONE PROPIONATE 25mcg/250mcg CFC-	6	c65W	FLIXOTIDE 500micrograms Accuhaler	2
c1Dx	SALMETEROL+FLUTICASONE PROPIONATE 50mcg/100mcg b-act	6	c65X	FLUTICASONE PROPIONATE 0.5mg/2mL nebulisation units	19
c1Dy	SALMETEROL+FLUTICASONE PROPIONATE 50mcg/250mcg b-act	6	c65Y	FLUTICASONE PROPIONATE 2mg/2mL nebulisation units	19
c1Dz	SALMETEROL+FLUTICASONE PROPIONATE 50mcg/500mcg b-act	6	c65Z	FLIXOTIDE 0.5mg/2mL Nebules	19
c1E	SALBUTAMOL [INHALATION PREPRATIONS 2]	1	c66	BECLOMETASONE DIPROPIONATE [RESPIRATORY USE 2]	2
c1E1	SALAMOL EASI-BREATHE 100mcg CFC-free breath-act aerosol inh	1	c661	*BDP 250micrograms Spacehaler	2
c1E2	PULVINAL SALBUTAMOL 200mcg breath-act dry powder inhaler	1	c662	BECOTIDE 50 EASI-BREATHE inhaler	2
c1E3	VENTODISKS 200micrograms diskhaler 15x8	1	c663	BECOTIDE 100 EASI-BREATHE inhaler	2
c1E4	VENTODISKS 400micrograms diskhaler 15x8	1	c664	BECLOFORTE EASI-BREATHE 250micrograms inhaler	2
c1E5	VENTODISKS 200micrograms disk refill 15x8	1	c665	QVAR 50 inhaler	2
c1E6	VENTODISKS 400micrograms disk refill 15x8	1	c666	QVAR 100 inhaler	2

Table S4: General Practice prescription codes for Asthma (cont)

General Practice Read Code Version 2 Asthma prescriptions (list from the Asthma in Swansea study 2009[24]) that have been categorised into diagnosis only, intermittent bronchodilator, persistent mild, persistent moderate and persistent severe prescription types (update extracted 2017).

Read Code v2	Description	Drug category	Read Code v2	Description	Drug category
c1E7	EASYHALER SALBUTAMOL 100mcg breath-actuated dry powder inh	1	c667	QVAR 50 Autohaler	2
c1E8	EASYHALER SALBUTAMOL 200mcg breath-actuated dry powder inh	1	c668	QVAR 100 Autohaler	2
c1E9	SALBULIN NOVOLIZER 100micrograms cartridge and inhaler	1	c669	BECLAZONE 200 inhaler	2
c1EA	SALBUTAMOL 100micrograms dry powder cartridge and inhaler	1	c66A	BECLOMETASONE DIPROP 50mcg breath-act dry powder inhaler	2
c1EB	SALBULIN NOVOLIZER 100micrograms dry powder cartridge refill	1	c66a	QVAR EASI-BREATHE 100mcg CFC-free breath-act dry pdr inhaler	2
c1EC	SALBUTAMOL 100micrograms dry powder cartridge refill	1	c66B	BECLOMETASONE DIPROP 100mcg breath-act dry powder inhaler	2
c1ED	VENTOLIN 50mg/10mL respirator solution	1	c66b	EASYHALER BECLOMETASONE 200mcg breath-act dry powder	2
c1EE	SALBUTAMOL 50mg/10mL respirator solution	17	c66C	BECLOMETASONE DIPROP 250mcg breath-act dry powder inhaler	2
c2	OTHER ADRENOCEPTOR STIMULANTS	1	c66c	CLENIL MODULITE 50micrograms CFC-free inhaler	2
c21	ADRENALINE [RESP]	5	c66D	ASMABEC 50micrograms Clickhaler	2
c211	ADRENALINE 500microgram/0.5mL injection	5	c66d	CLENIL MODULITE 100micrograms CFC-free inhaler	2
c212	*ADRENALINE 1mg/1mL injection	5	c66E	ASMABEC 100micrograms Clickhaler	2
c213	MEDIHALER-EPI 280micrograms inhaler	5	c66e	CLENIL MODULITE 200micrograms CFC-free inhaler	2
c214	MIN-I-JET ADREN 500microgram/0.5mL injection	5	c66F	ASMABEC 250micrograms Clickhaler	2
c215	MIN-I-JET ADREN 1mg/1mL injection	5	c66f	CLENIL MODULITE 250micrograms CFC-free inhaler	2
c216	ADRENALINE 280micrograms inhaler	5	c66G	BECLOMETASONE DIPROP 400mcg breath-act dry powder inhaler	2
c22	EPHEDRINE HYDROCHLORIDE [RESPIRATORY USE]	1	c66g	BECLOMETASONE DIPROPIONATE 200micrograms CFC-free	2
c221	EPHEDRINE HYDROCHLORIDE 15mg tablets	1	c66H	BECLOMETASONE DIPROP 200mcg breath-act dry powder inhaler	2
c222	EPHEDRINE HYDROCHLORIDE 30mg tablets	1	c66h	BECLOMETASONE DIPROPIONATE 250micrograms CFC-free	2
c223	EPHEDRINE HYDROCHLORIDE 60mg tablets	1	c66I	PULVINAL BECLOMETHASONE DIPROP 100mcg breath-act dry pdr	2
c224	EPHEDRINE HYDROCHLORIDE 15mg/5mL elixir	1	c66J	PULVINAL BECLOMETHASONE DIPROP 200mcg breath-act dry pdr	2
c225	*CAM SF 15mg/5mL mixture	1	c66K	PULVINAL BECLOMETHASONE DIPROP 400mcg breath-act dry pdr	2
c226	CAM 4mg/5mL sugar free mixture	1	c66L	BECLOMETASONE 100 Cyclocaps	2
c227	EPHEDRINE HYDROCHLORIDE 4mg/5mL sugar free mixture	1	c66M	BECLOMETASONE 200 Cyclocaps	2
c23	*ISOETHARINE HYDROCHLORIDE	1	c66N	BECLOMETASONE 400 Cyclocaps	2
c231	*NUMOTAC 10mg m/r tablets	1	c66P	BECODISK 100micrograms diskhaler 15x8	2
c23z	ISOETHARINE HCL 10mg m/r tablets	1	c66Q	BECODISK 200micrograms diskhaler 15x8	2
c24	ISOPRENALINE SULPHATE	1	c66R	BECODISK 400micrograms diskhaler 15x8	2
c241	*ALEUDRIN 20mg tablets	1	c66S	BECODISK 100micrograms disk refill 15x8	2
c242	ALEUDRIN 1% spray for nebuliser	17	c66T	BECODISK 200micrograms disk refill 15x8	2
c243	ISO-AUTOHALER 80microgram inhaler	1	c66U	BECODISK 400micrograms disk refill 15x8	2
c244	ISO-AUTOHALER 80microgram inhaler	1	c66V	BECLOMETASONE DIPROPIONATE 50micrograms CFC-free inhaler	2
c245	MEDIHALER-ISO 80micrograms inhaler	1	c66W	BECLOMETASONE DIPROPIONATE 100micrograms CFC-free	2
c246	MEDIHALER-ISO FORTE 400micrograms inhaler	1	c66X	BECLOMETASONE DIPROPIONATE 50mcg CFC-free br-act inhaler	2
c24v	ISOPRENALINE SULPHATE 20mg tablets	1	c66Y	BECLOMETASONE DIPROPIONATE 100mcg CFC-free br-act inhaler	2
c24w	ISOPRENALINE SULPHATE 1% spray for nebuliser	17	c66Z	QVAR EASI-BREATHE 50mcg CFC-free breath-act dry pdr inhaler	2
c24x	ISOPRENALINE SULPHATE 80micrograms inhaler	1	c67	BUDESONIDE+FORMOTEROL	6
c24y	ISOPRENALINE SULPHATE 80micrograms inhaler refill	1	c671	SYMBICORT 100/6 Turbohaler	6
c24z	ISOPRENALINE SULPHATE 400micrograms inhaler	1	c672	SYMBICORT 200/6 Turbohaler	6

Table S4: General Practice prescription codes for Asthma (cont)

General Practice Read Code Version 2 Asthma prescriptions (list from the Asthma in Swansea study 2009[24]) that have been categorised into diagnosis only, intermittent bronchodilator, persistent mild, persistent moderate and persistent severe prescription types (update extracted 2017).

Read Code v2	Description	Drug category	Read Code v2	Description	Drug category
c25	ORCIPRENALINE SULPHATE [RESPIRATORY USE]	1	c673	SYMBICORT 400/12 Turbohaler	6
c251	*ALUPENT 20mg tablets	1	c674	DUORESP SPIROMAX 160mcg/4.5mcg breath-act dry powder inhaler	6
c252	ALUPENT 10mg/5mL syrup	1	c675	DUORESP SPIROMAX 320mcg/9mcg breath-act dry powder inhaler	6
c253	ALUPENT 500microgram/1mL injection	5	c67x	BUDESONIDE+FORMOTEROL FUMARATE 400/12mcg b-act dry pdr	6
c254	*ALUPENT 750micrograms inhaler	1	c67y	BUDESONIDE+FORMOTEROL FUMARATE 200/6mcg bth-act dry	6
c255	ALUPENT 750micrograms inhaler refill	1	c67z	BUDESONIDE+FORMOTEROL FUMARATE 100/6mcg bth-act dry	6
c25v	*ORCIPRENALINE 20mg tablets	1	c68	MOMETASONE [RESPIRATORY USE]	2
c25w	ORCIPRENALINE 10mg/5mL syrup	1	c681	MOMETASONE FUROATE 200mcg breath-act dry powder inhaler	2
c25x	ORCIPRENALINE 500microgram/1mL injection	5	c682	MOMETASONE FUROATE 400mcg breath-act dry powder inhaler	2
c25y	ORCIPRENALINE 750micrograms inhaler	1	c683	ASMANEX TWISTHALER 200mcg breath-act dry powder inhaler	2
c25z	ORCIPRENALINE 750micrograms inhaler refill	1	c684	ASMANEX TWISTHALER 400mcg breath-act dry powder inhaler	2
c3	ANTICHOLINERGIC BRONCHODILATORS	1	c69	CICLESONIDE	2
c31	IPRATROPIUM BROMIDE [1]	1	c691	ALVESCO 160micrograms inhaler	2
c311	*ATROVENT 20micrograms inhaler	1	c692	ALVESCO 80micrograms inhaler	2
c312	ATROVENT 500microgram/2mL nebuliser solution	17	c69y	CICLESONIDE 80micrograms inhaler	2
c313	ATROVENT FORTE 40microgram inhaler	1	c69z	CICLESONIDE 160micrograms inhaler	2
c314	ATROVENT 250microgram/1mL nebuliser solution	17	c6A	BECLOMETASONE+FORMOTEROL	6
c315	ATROVENT 20micrograms Autohaler	1	c6A1	FOSTAIR 100micrograms/6micrograms inhaler	6
c316	STERI-NEB IPRATROPIUM 250micrograms/1mL nebulisation units	17	c6A2	FOSTAIR NEXTHALER 100micrograms/6micrograms powder inhaler	6
c317	STERI-NEB IPRATROPIUM 500micrograms/2mL nebulisation units	17	c6Ay	BECLOMET DIPROP+FORMOTERL FUMARATE DIHYD	6
c318	ATROVENT 40micrograms Aerocaps refill pack	1	c6Az	BECLOMETASONE+FORMOTEROL 100micrograms/6micrograms	6
c319	ATROVENT 40micrograms Aerocaps+Aerohaler device	1	c6B	FLUTICASONE+VILANTEROL	6
c31A	IPRATROPIUM BROMIDE 40mcg inhalation capsules	1	c6B1	RELVAR ELLIPTA 184micrograms/22micrograms inhaler	6
c31B	IPRATROPIUM BROMIDE 40mcg inhalation capsules+inhaler device	1	c6B2	FLUTICASONE FUROATE+VILANTEROL 184mcg/22mcg dry pdr	6
c31C	RESPONTIN 250micrograms/1mL Nebules	17	c6B3	RELVAR ELLIPTA 92micrograms/22micrograms inhaler	6
c31D	RESPONTIN 500micrograms/2mL Nebules	17	c6B4	FLUTICASONE FUROATE+VILANTEROL 92mcg/22mcg dry pdr	6
c31E	TROPIOVENT 250micrograms/1mL Steripoules	17	c7	ASTHMA PROPHYLAXIS	1
c31F	TROPIOVENT 500micrograms/2mL Steripoules	17	c71	SODIUM CROMOGLICATE [ASTHMA]	13
c31G	ATROVENT 20micrograms CFC-free inhaler	1	c711	*INTAL 1mg inhaler	13
c31t	IPRATROPIUM BROMIDE 20micrograms CFC-free inhaler	1	c712	*INTAL HALERMATIC insufflator	13
c31u	IPRATROPIUM 20micrograms breath-actuated aerosol inhaler	1	c713	INTAL 20mg spincaps	13
c31v	IPRATROPIUM 250micrograms/1mL nebuliser solution	17	c714	INTAL SPINHALER insufflator	13
c31w	IPRATROPIUM 500micrograms/2mL nebuliser solution	17	c715	INTAL 20mg/2mL nebuliser solution	20
c31x	IPRATROPIUM 20micrograms inhaler	1	c716	*INTAL 5mg inhaler	13
c31y	IPRATROPIUM 250micrograms/mL nebuliser solution	17	c717	SODIUM CROMOGLICATE 20mg inhalation capsules	20
c31z	IPRATROPIUM 40microgram inhaler	1	c718	SODIUM CROMOGLICATE 20mg/2mL nebuliser solution	20
c32	OXITROPIUM BROMIDE	1	c719	SODIUM CROMOGLICATE 5mg inhaler	13
c321	OXITROPIUM 100micrograms/dose inhaler 200dose	1	c71a	*INTAL 5mg Autohaler	13
c322	OXIVENT 100micrograms/dose inhaler 200dose	1	c71b	STERI-NEB CROMOGEN 20mg nebulisation units	20

Table S4: General Practice prescription codes for Asthma (cont)

General Practice Read Code Version 2 Asthma prescriptions (list from the Asthma in Swansea study 2009[24]) that have been categorised into diagnosis only, intermittent bronchodilator, persistent mild, persistent moderate and persistent severe prescription types (update extracted 2017).

Read Code v2	Description	Drug category	Read Code v2	Description	Drug category
c323	OXIVENT 100micrograms Autohaler	1	c71c	CROMOGEN 5mg inhaler	13
c324	OXITROPIUM 100micrograms breath-actuated aerosol inhaler	1	c71d	INTAL FISONAIR 5mg inhaler + spacer device	13
c33	TIOTROPIUM	7	c71e	SODIUM CROMOGLICATE 5mg inhaler + spacer device	13
c331	TIOTROPIUM 18micrograms inhalation capsules	7	c71f	SODIUM CROMOGLYCATE 5mg auto inhaler	13
c332	TIOTROPIUM 18micrograms capsules with inhaler device	7	c71g	INTAL SYNCRONER 5mg inhaler + spacer device 2x112dose	13
c333	TIOTROPIUM 2.5micrograms inhalation carts+inhaler device	7	c71h	SODIUM CROMOGLICATE 5mg breath-actuated aerosol inhaler	13
c33x	SPIRIVA RESPIMAT 2.5mcg cartridges+Respimat inhaler device	7	c71i	INTAL 5mg CFC-free inhaler	13
c33y	SPIRIVA COMBOPACK 18mcg caps+HandiHaler inhaler device	7	c71j	CROMOGEN EASI-BREATHE 5mg breath-actuated aerosol inhaler	13
c33z	SPIRIVA 18micrograms inhalation capsules	7	c71k	SODIUM CROMOGLICATE 5mg CFC-free inhaler	13
c4	XANTHINE BRONCHODILATORS	8	c72	SODIUM CROMOGLICATE COMPOUNDS	13
c41	AMINOPHYLLINE	8	c721	*INTAL COMPOUND spincaps	13
c411	AMINOPHYLLINE 100mg tablets	8	c722	*AEROCROM inhaler	11
c412	AMINOPHYLLINE 250mg/10mL injection	5	c723	AEROCROM SYNCRONER inhaler + spacer device	11
c413	AMINOPHYLLINE 500mg/2mL injection	5	c72y	SODIUM CROMOGLICATE+SALBUTAMOL 1mg/100mcg inhaler +	11
c414	AMINOPHYLLINE 50mg suppositories	8	c72z	SODIUM CROMOGLICATE+SALBUTAMOL 1mg/100micrograms	11
c415	AMINOPHYLLINE 100mg suppositories	8	c73	KETOTIFEN [ASTHMA PROPHYLAXIS]	12
c416	AMINOPHYLLINE 150mg suppositories	8	c731	*ZADITEN 1mg capsules	12
c417	AMINOPHYLLINE 180mg suppositories	8	c732	ZADITEN 1mg tablets	12
c418	AMINOPHYLLINE 360mg suppositories	8	c733	ZADITEN 1mg/5mL elixir	12
c419	*THEODROX tablets	8	c734	*KETOTIFEN 1mg capsules	12
c41A	*NORPHYLLIN 100mg tablets	8	c735	KETOTIFEN 1mg tablets	12
c41a	PHYLLOCONTIN CONTINUS 225mg m/r tablets	26	c736	KETOTIFEN 1mg/5mL elixir	12
c41B	NORPHYLLIN SR 225mg m/r tablets	26	c73x	*KETOTIFEN 1mg capsules	12
c41b	PHYLLOCONTIN FORTE 350mg m/r tablets	26	c73y	*KETOTIFEN 1mg tablets	12
c41C	NORPHYLLIN SR 350mg m/r tablets	26	c73z	*KETOTIFEN 1mg/5mL elixir	12
c41c	PHYLLOCONTIN PAEDIATRIC 100mg m/r tablets	26	c74	NEDOCROMIL SODIUM [ASTHMA]	13
c41d	AMINOPHYLLINE 225mg m/r tablets	26	c741	*TILADE MINT 2mg inhaler	13
c41e	*PECRAM 225mg m/r tablets	26	c742	*NEDOCROMIL SODIUM 2mg inhaler	13
c41f	AMINOPHYLLINE 350mg m/r tablets	26	c743	*TILADE MINT 2mg inhaler	13
c41g	AMINOPHYLLINE 100mg m/r tablets	26	c744	TILADE MINT SYNCRONER 2mg inhaler	13
c41h	*AMNIVENT 225mg m/r tablets	26	c745	NEDOCROMIL SODIUM 2mg inhaler + spacer	13
c41i	*AMNIVENT 350mg m/r tablets	26	c746	NEDOCROMIL SODIUM 2mg CFC-free inhaler	13
c41j	MIN-I-JET AMINOPHYLLINE 250mg/10mL injection	5	c747	TILADE 2mg CFC-free inhaler	13
c41k	AMINOPHYLLINE 250mg/10mL prefilled syringe	5	cA	LEUKOTRIENE RECEPTOR ANTAGONIST	4
c41m	AMINOPHYLLINE HYDRATE 225mg m/r tablets	26	cA1	MONTELUKAST	4
c42	CHOLINE THEOPHYLLINATE	8	cA11	MONTELUKAST 10mg tablets	4
c421	*CHOLEDYL 100mg tablets	8	cA12	MONTELUKAST 5mg chewable tablets	4
c422	*CHOLEDYL 200mg tablets	8	cA13	SINGULAIR 10mg tablets	4
c423	*CHOLEDYL 62.5mg/5mL syrup	8	cA14	SINGULAIR PAEDIATRIC 5mg chewable tablets	4

Evans A, *et al. Thorax* 2021; 76:116–125. doi: 10.1136/thoraxjnl-2020-215422

Table S4: General Practice prescription codes for Asthma (cont)

General Practice Read Code Version 2 Asthma prescriptions (list from the Asthma in Swansea study 2009[24]) that have been categorised into diagnosis only, intermittent bronchodilator, persistent mild, persistent moderate and persistent severe prescription types (update extracted 2017).					
**Read Code v2**	**Description**	**Drug category**	**Read Code v2**	**Description**	**Drug category**
c424	SABIDAL SR-270 424mg m/r tablets	26	cA15	SINGULAIR PAEDIATRIC 4mg chewable tablets	4
c42w	CHOLINE THEOPHYLLINATE 100mg tablets	8	cA16	SINGULAIR PAEDIATRIC 4mg/sachet granules	4
c42x	CHOLINE THEOPHYLLINATE 200mg tablets	8	cA1y	MONTELUKAST 4mg/sachet granules	4
c42y	CHOLINE THEOPHYLLINATE 62.5mg/5mL syrup	8	cA1z	MONTELUKAST 4mg chewable tablets	4
c42z	CHOLINE THEOPHYLLINATE 424mg m/r tablets	26	cA2	ZAFIRLUKAST	4
c43	THEOPHYLLINE	8	cA21	ZAFIRLUKAST 20mg tablets	4
c431	*BIOPHYLLINE 125mg/5mL syrup	8	cA22	ACCOLATE 20mg tablets	4
c432	*NUELIN 125mg tablets	8	ck1	OMALIZUMAB	21
c433	*NUELIN 60mg/5mL liquid	8	ck11	OMALIZUMAB 150mg injection(pdr for recon)+solvent	21
c434	*LASMA 300mg m/r tablets	4	ck12	XOLAIR 150mg injection(pdr for recon)+solvent	21
c435	NUELIN SA 175mg m/r tablets	26	ck13	OMALIZUMAB 75mg/0.5mL soln for injection prefilled syringe	21
c436	NUELIN SA-250 250mg m/r tablets	26	ck14	XOLAIR 75mg/0.5mL solution for injection prefilled syringe	21
c437	*PRO-VENT 300mg m/r capsules	26	ck15	OMALIZUMAB 150mg/1mL soln for injection prefilled syringe	21
c438	SLO-PHYLLIN 60mg m/r capsules	26	ck16	XOLAIR 150mg/1mL solution for injection prefilled syringe	21
c439	SLO-PHYLLIN 125mg m/r capsules	26			

Evans A, *et al. Thorax* 2021; 76:116–125. doi: 10.1136/thoraxjnl-2020-215422

Table S5: General Practice prescription codes for Endocrine corticosteroids

General Practice Corticosteroid Read codes v2 for the Endocrine system (extraction 2017)	
**Endocrine corticosteroids tablets or oral solution**	
**Code**	**Description**
fe1z.	BETAMETHASONE 4mg/1mL injection
fe3A.	DEXSOL 2mg/5mL oral solution
fe3B.	DEXAMETHASONE 10mg/5mL oral solution
fe3C.	MARTAPAN 2mg/5mL oral solution
fe3r.	DEXAMETHASONE 500micrograms/5mL solution
fe3s.	DEXAMETHASONE 2mg/5mL sugar free solution
fe3u.	DEXAMETHASONE 2mg/5mL liquid
fe41.	HYDROCORTISONE 10mg tablets
fe42.	HYDROCORTISONE 20mg tablets
fe43.	*HYDROCORTISTAB 20mg tablets
fe44.	*HYDROCORTONE 10mg tablets
fe45.	*HYDROCORTONE 20mg tablets
fe51.	MEDRONE 2mg tablets
fe52.	MEDRONE 4mg tablets
fe53.	MEDRONE 16mg tablets
fe5f.	MEDRONE 100mg tablets
fe5m.	METHYLPREDNISOLONE 100mg tablets
fe5n.	METHYLPREDNISOLONE 2mg tablets
fe5o.	METHYLPREDNISOLONE 4mg tablets
fe5p.	METHYLPREDNISOLONE 16mg tablets
fe61.	PREDNISOLONE 1mg tablets
fe62.	PREDNISOLONE 5mg tablets
fe64.	*DELTA-PHORICOL 5mg tablets
fe65.	DELTACORTRIL ENTERIC 2.5mg tablets
fe66.	DELTACORTRIL ENTERIC 5mg tablets
fe67.	*DELTALONE 1mg tablets
fe68.	*DELTALONE 5mg tablets
fe69.	*DELTASTAB 1mg tablets
fe6a.	*DELTASTAB 5mg tablets
fe6c.	*PRECORTISYL 1mg tablets
fe6d.	*PRECORTISYL 5mg tablets
fe6e.	PRECORTISYL FORTE 25mg tablets
fe6f.	*PREDNESOL 5mg tablets
fe6g.	*SINTISONE 5mg tablets
fe6h.	PREDNISOLONE 2.5mg e/c tablets
fe6i.	PREDNISOLONE 5mg e/c tablets
fe6j.	PREDNISOLONE 5mg soluble tablets
fe6k.	PREDNISOLONE 50mg tablets
fe6l.	DILACORT 5mg gastro-resistant tablets
fe6m.	DILACORT 2.5mg gastro-resistant tablets
fe6n.	PEVANTI 2.5mg tablets
fe6o.	PEVANTI 25mg tablets
fe6p.	PEVANTI 5mg tablets
fe6q.	PEVANTI 10mg tablets
fe6r.	PEVANTI 20mg tablets
fe6s.	PREDNISOLONE 20mg tablets
fe6t.	PREDNISOLONE 10mg tablets
fe6v.	PREDNISOLONE 2.5mg tablets
fe6w.	*PREDNISOLONE 2.5mg tablets
fe71.	*PREDNISONE 1mg tablets
fe72.	*PREDNISONE 5mg tablets
fe73.	*DECORTISYL 5mg tablets
fe74.	*ECONOSONE 1mg tablets
fe75.	*ECONOSONE 5mg tablets
fe81.	*TRIAMCINOLONE 2mg tablets
fe82.	*TRIAMCINOLONE 4mg tablets
fe86.	*LEDERCORT 2mg tablets
fe87.	*LEDERCORT 4mg tablets
fe91.	DEFLAZACORT 6mg tablets
fe92.	CALCORT 6mg tablets
fe93.	*DEFLAZACORT 30mg tablets
fe94.	*CALCORT 30mg tablets
fe95.	*DEFLAZACORT 1mg tablets
fe96.	*CALCORT 1mg tablets

Evans A, *et al. Thorax* 2021; 76:116–125. doi: 10.1136/thoraxjnl-2020-215422

Table S5: General Practice prescription codes for Endocrine corticosteroids (cont)

General Practice Corticosteroid Read codes v2 for the Endocrine system (extraction 2017)	
**Endocrine Corticosteroids generic terms (assumed as tablets or liquid)**	
**Code**	**Description**
fe3..	DEXAMETHASONE [ENDOCRINE]
fe4..	HYDROCORTISONE
fe5..	METHYLPREDNISOLONE [ENDOCRINE]
fe6..	PREDNISOLONE [ENDOCRINE]
fe7..	PREDNISONE
fe8..	TRIAMCINOLONE [ENDOCRINE]
fe9..	DEFLAZACORT
**Endocrine corticosteroid injections**	
**Code**	**Description**
fe34.	*DECADRON 8mg/2mL injection
fe35.	DECADRON SHOCK-PAK 100mg/5mL injection
fe38.	*ORADEXON 5mg/1mL injection
fe39.	*ORADEXON 10mg/2mL injection
fe3D.	DEXAMETHASONE 3.8mg/1mL solution for injection
fe3p.	DEXAMETHASONE 6.6mg/2mL solution for injection
fe3q.	DEXAMETHASONE 3.3mg/1mL solution for injection
fe3w.	DEXAMETHASONE 8mg/2mL injection
fe3x.	DEXAMETHASONE 100mg/5mL injection
fe3z.	DEXAMETHASONE 4mg/1mL injection
fe46.	HYDROCORTISONE 100mg injection
fe47.	HYDROCORTISONE 500mg injection
fe48.	EFCORTELAN SOLUBLE 100mg injection
fe49.	EFCORTESOL 100mg/1mL injection
fe4a.	EFCORTESOL 500mg/5mL injection
fe4b.	SOLU-CORTEF+WATER 100mg injection
fe4c.	SOLU-CORTEF 100mg injection
fe4d.	HYDROCORTISONE 100mg/1mL injection
fe54.	MIN-I-MIX METHYLPREDNIS. 500mg injection
fe55.	MIN-I-MIX METHYLPREDNISOLONE 1g injection
fe56.	SOLU-MEDRONE 40mg injection powder+diluent
fe57.	SOLU-MEDRONE 125mg injection powder+diluent
fe58.	SOLU-MEDRONE 500mg injection powder+diluent
fe59.	SOLU-MEDRONE 1g injection powder+diluent
fe5a.	SOLU-MEDRONE 2g injection
fe5b.	DEPO-MEDRONE 40mg/1mL injection
fe5c.	DEPO-MEDRONE 80mg/2mL injection
fe5d.	*DEPO-MEDRONE 80mg syringe
fe5e.	DEPO-MEDRONE 120mg/3mL injection
fe5g.	METHYLPREDNISOLONE 500mg injection (pdr for recon)
fe5h.	METHYLPREDNISOLONE 1g injection (pdr for recon)
fe5q.	METHYLPREDNISOLONE 500mg injection
fe5r.	METHYLPREDNISOLONE 1g injection
fe5s.	METHYLPREDNISOLONE 40mg injection powder+diluent
fe5t.	METHYLPREDNISOLONE 125mg injection powder+diluent
fe5u.	METHYLPREDNISOLONE 500mg injection powder+diluent
fe5v.	METHYLPREDNISOLONE 1g injection powder+diluent
fe5w.	METHYLPREDNISOLONE 2g injection powder+diluent
fe5x.	METHYLPREDNISOLONE 40mg/1mL injection
fe5y.	METHYLPREDNISOLONE 80mg/2mL injection
fe5z.	METHYLPREDNISOLONE 200mg/5mL injection
fe63.	*CODELSOL 32mg/2mL injection
fe6b.	DELTASTAB 25mg/1mL injection
fe6u.	PREDNISOLONE 32mg/2mL injection
fe6y.	PREDNISOLONE 125mg/5mL injection
fe6z.	PREDNISOLONE 25mg tablets
fe83.	KENALOG 40mg/mL injection
fe84.	*KENALOG 40mg/1mL syringe
fe85.	*KENALOG 80mg/2mL injection
fe88.	KENALOG 80mg/2mL i-m prefilled syringe
fe8u.	TRIAMCINOLONE ACETONIDE 40mg/1mL intramuscular injection
fe8v.	TRIAMCINOLONE ACETONIDE 80mg/2mL i-m prefilled syringe
fe8w.	TRIAMCINOLONE ACETONIDE 40mg/1mL i-m prefilled syringe
fe8x.	TRIAMCINOLONE 40mg/1mL injection
fe8y.	TRIAMCINOLONE 80mg/2mL injection

Evans A, *et al. Thorax* 2021; 76:116–125. doi: 10.1136/thoraxjnl-2020-215422

Table S6: General Practice respiratory diagnoses

General Practice Respiratory diagnosis categories for Read codes v2 (extraction 2017)	
**Upper respiratory tract infection**	
**Code**	**Description**
H0...	Acute respiratory infections
H00..	Acute nasopharyngitis
H01..	Acute sinusitis
H010.	Acute maxillary sinusitis
H011.	Acute frontal sinusitis
H012.	Acute ethmoidal sinusitis
H01y.	Other acute sinusitis
H01z.	Acute sinusitis NOS
H02..	Acute pharyngitis
H021.	Acute phlegmonous pharyngitis
H022.	Acute ulcerative pharyngitis
H023.	Acute bacterial pharyngitis
H023z	Acute bacterial pharyngitis NOS
H024.	Acute viral pharyngitis
H02z.	Acute pharyngitis NOS
H03..	Acute tonsillitis
H030.	Acute erythematous tonsillitis
H031.	Acute follicular tonsillitis
H032.	Acute ulcerative tonsillitis
H033.	Acute catarrhal tonsillitis
H035.	Acute bacterial tonsillitis
H0351	Acute staphylococcal tonsillitis
H035z	Acute bacterial tonsillitis NOS
H036.	Acute viral tonsillitis
H037.	Recurrent acute tonsillitis
H03z.	Acute tonsillitis NOS
H04..	Acute laryngitis and tracheitis
H040.	Acute laryngitis
H0402	Acute catarrhal laryngitis
H040w	Acute viral laryngitis unspecified
H040z	Acute laryngitis NOS
H041.	Acute tracheitis
H0410	Acute tracheitis without obstruction
H041z	Acute tracheitis NOS
H042.	Acute laryngotracheitis
H0420	Acute laryngotracheitis without obstruction
H042z	Acute laryngotracheitis NOS
H043.	Acute epiglottitis (non strep)
H0432	Acute obstructive laryngitis
H043z	Acute epiglottitis NOS
H04z.	Acute laryngitis and tracheitis NOS
H05..	Other acute upper respiratory infections
H050.	Acute laryngopharyngitis
H051.	Acute upper respiratory tract infection
H052.	Pharyngotracheitis
H053.	Tracheopharyngitis
H054.	Recurrent upper respiratory tract infection
H055.	Pharyngolaryngitis
H05y.	Other upper respiratory infections of multiple sites
H05z.	Upper respiratory infection NOS
H15..	Peritonsillar abscess – quinsy
H271.	Influenza with other respiratory manifestation
H2710	Influenza with laryngitis
H2711	Influenza with pharyngitis
H271z	Influenza with respiratory manifestations NOS
Hyu0.	[X]Acute upper respiratory infections
Hyu02	[X]Acute tonsillitis due to other specified organisms
**Influenza and pneumonia**	
**Code**	**Description**
H2...	Pneumonia and influenza
H20..	Viral pneumonia
H201.	Pneumonia due to respiratory syncytial virus
H202.	Pneumonia due to parainfluenza virus
H20y.	Viral pneumonia NEC

Table S6: General Practice respiratory diagnoses (cont)

**General Practice Respiratory diagnosis categories for Read codes v2 (extraction 2017)**	
**Influenza and pneumonia (cont)**	
**Code**	**Description**
H20z.	Viral pneumonia NOS
H21..	Lobar (pneumococcal) pneumonia
H22..	Other bacterial pneumonia
H222.	Pneumonia due to haemophilus influenzae
H223.	Pneumonia due to streptococcus
H224.	Pneumonia due to staphylococcus
H22y.	Pneumonia due to other specified bacteria
H22yz	Pneumonia due to bacteria NOS
H22z.	Bacterial pneumonia NOS
H23..	Pneumonia due to other specified organisms
H231.	Pneumonia due to mycoplasma pneumoniae
H23z.	Pneumonia due to specified organism NOS
H24..	Pneumonia with infectious diseases EC
H243.	Pneumonia with whooping cough
H25..	Bronchopneumonia due to unspecified organism
H26..	Pneumonia due to unspecified organism
H260.	Lobar pneumonia due to unspecified organism
H2600	Lung consolidation
H261.	Basal pneumonia due to unspecified organism
H27..	Influenza
H270.	Influenza with pneumonia
H2700	Influenza with bronchopneumonia
H270z	Influenza with pneumonia NOS
H27y1	Influenza with gastrointestinal tract involvement
H27z.	Influenza NOS
H28..	Atypical pneumonia
H2y..	Other specified pneumonia or influenza
H2z..	Pneumonia or influenza NOS
Hyu08	[X]Other viral pneumonia
Hyu0H	[X]Other pneumonia, organism unspecified
**Lower respiratory tract infection including bronchiolitis when coded with bronchitis**	
**Code**	**Description**
H06..	Acute bronchitis and bronchiolitis
H060.	Acute bronchitis
H0603	Acute purulent bronchitis
H0604	Acute croupous bronchitis
H0605	Acute tracheobronchitis
H0606	Acute pneumococcal bronchitis
H0609	Acute neisseria catarrhalis bronchitis
H060A	Acute bronchitis due to mycoplasma pneumoniae
H060E	Acute bronchitis due to rhinovirus
H060w	Acute viral bronchitis unspecified
H060x	Acute bacterial bronchitis unspecified
H060z	Acute bronchitis NOS
H062.	Acute lower respiratory tract infection
H06z.	Acute bronchitis or bronchiolitis NOS
H06z0	Chest infection NOS
H06z1	Lower resp tract infection
H06z2	Recurrent chest infection
H07..	Chest cold
H0y..	Other specified acute respiratory infections
H0z..	Acute respiratory infection NOS
H3...	Chronic obstructive pulmonary disease
H30..	Bronchitis unspecified
H300.	Tracheobronchitis NOS
H301.	Laryngotracheobronchitis
H302.	Wheezy bronchitis
H30z.	Bronchitis NOS
H3101	Smokers' cough
H3122	Acute exacerbation of chronic obstructive airways disease
Hyu10	[X]Acute bronchitis due to other specified organisms

Evans A, *et al. Thorax* 2021; 76:116–125. doi: 10.1136/thoraxjnl-2020-215422

Table S6: General Practice respiratory diagnoses (cont)

**General Practice Respiratory diagnosis categories for Read codes v2 (extraction 2017)**	
**Bronchiolitis**	
**Code**	**Description**
H061.	Acute bronchiolitis
H0612	Acute bronchiolitis with bronchospasm
H0613	Acute exudative bronchiolitis
H0615	Acute bronchiolitis due to respiratory syncytial virus
H061z	Acute bronchiolitis NOS
Hyu20	[X]Other seasonal allergic rhinitis
Hz...	Respiratory system diseases NOS
**Chronic upper respiratory disease**	
**Code**	**Description**
H025.	Allergic pharyngitis
H1...	Other upper respiratory tract diseases
H10..	Deviated nasal septum – acquired
H11..	Nasal polyps
H110.	Polyp of nasal cavity
H110z	Polyp of nasal cavity NOS
H11z.	Nasal polyp NOS
H12..	Chronic pharyngitis and nasopharyngitis
H120.	Chronic rhinitis
H1200	Chronic simple rhinitis
H1201	Chronic catarrhal rhinitis
H1202	Chronic hypertrophic rhinitis
H120z	Chronic rhinitis NOS
H121.	Chronic pharyngitis
H1210	Simple chronic pharyngitis
H1211	Atrophic pharyngitis
H1212	Granular pharyngitis
H122.	Chronic nasopharyngitis
H13..	Chronic sinusitis
H130.	Chronic maxillary sinusitis
H131.	Chronic frontal sinusitis
H135.	Recurrent sinusitis
H13z.	Chronic sinusitis NOS
H14..	Chronic tonsil and adenoid disease
H140.	Chronic tonsillitis
H141.	Tonsil and/or adenoid hypertrophy
H1410	Hypertrophy of tonsils and adenoids
H1411	Hypertrophy of tonsils alone
H1412	Hypertrophy of adenoids alone
H141z	Hypertrophy of tonsils and adenoids NOS
H143.	Chronic adenotonsillitis
H14y4	Tonsil ulcer
H14y7	Cyst of tonsil
H14z0	Chronic tonsil disease NOS
H160.	Chronic laryngitis
H1601	Chronic catarrhal laryngitis
H161.	Chronic laryngotracheitis
H17..	Allergic rhinitis
H170.	Allergic rhinitis due to pollens
H171.	Allergic rhinitis due to other allergens
H1710	Allergy to animal
H1711	Dog allergy
H172.	Allergic rhinitis due to unspecified allergen
H17z.	Allergic rhinitis NOS
H18..	Vasomotor rhinitis
H1y..	Other specified diseases of upper respiratory tract
H1y0.	Nasal turbinate hypertrophy
H1y1.	Other nasal cavity and sinus disease
H1y10	Nasal septum abscess
H1y12	Nasal septum ulcer
H1y16	Nasal obstruction
H1y1z	Nasal cavity and sinus disease NOS
H1y2.	Other pharyngeal disease NEC
H1y22	Parapharyngeal abscess
H1y23	Retropharyngeal abscess

Table S6: General Practice respiratory diagnoses (cont)

General Practice Respiratory diagnosis categories for Read codes v2 (extraction 2017)	
**Chronic upper respiratory disease (cont)**	
**Code**	**Description**
H1y2z	Other pharyngeal disease NOS
H1y3.	Paralysis of vocal cords or larynx
H1y56	Vocal cord nodule
H1y73	Stenosis of larynx
H1y74	Laryngeal spasm
H1y77	Obstruction of larynx NOS
H1y7B	Laryngomalacia
H1yz.	Other upper respiratory tract diseases NOS
H1z..	Upper respiratory tract disease NOS
H3123	Bronchiolitis obliterans
H31y0	Chronic tracheitis
H5B..	Sleep apnoea
H5B0.	Obstructive sleep apnoea
H5C..	Choking due to airways obstruction
H5y04	Tracheo-oesophageal fistula following tracheostomy
Hy...	Other specified diseases of respiratory system
**Chronic lower respiratory disease**	
**Code**	**Description**
H263.	Pneumonitis, unspecified
H34..	Bronchiectasis
H34z.	Bronchiectasis NOS
H35..	Extrinsic allergic alveolitis
H357.	Ventilation pneumonitis
H4...	Lung disease due to external agents
H462.	Upper respiratory inflammation due to chemical fumes
H47..	Pneumonitis due to inhalation of solids or liquids
H470.	Pneumonitis due to inhalation of food or vomitus
H5...	Other respiratory system diseases
H50..	Empyema
H51..	Pleurisy
H51z.	Pleural effusion NOS
H51zz	Pleural effusion NOS
H52..	Pneumothorax
H520.	Spontaneous tension pneumothorax
H52y.	Other spontaneous pneumothorax
H52yz	Other spontaneous pneumothorax NOS
H52z.	Pneumothorax NOS
H541.	Pulmonary congestion
H541z	Pulmonary oedema NOS
H58..	Other diseases of lung
H580.	Pulmonary collapse with atelectasis
H581.	Interstitial emphysema
H58y0	Broncholithiasis
H58z.	Lung disease NOS
H59..	Respiratory failure
H590.	Acute respiratory failure
H5yy.	Other diseases of respiratory system NEC
H5yz.	Other diseases of respiratory system NOS
H5z..	Respiratory system diseases NOS
**Unspecified respiratory illness**	
**Code**	**Description**
H....	Respiratory system diseases
**Croup**	
**Code**	**Description**
H044.	Croup

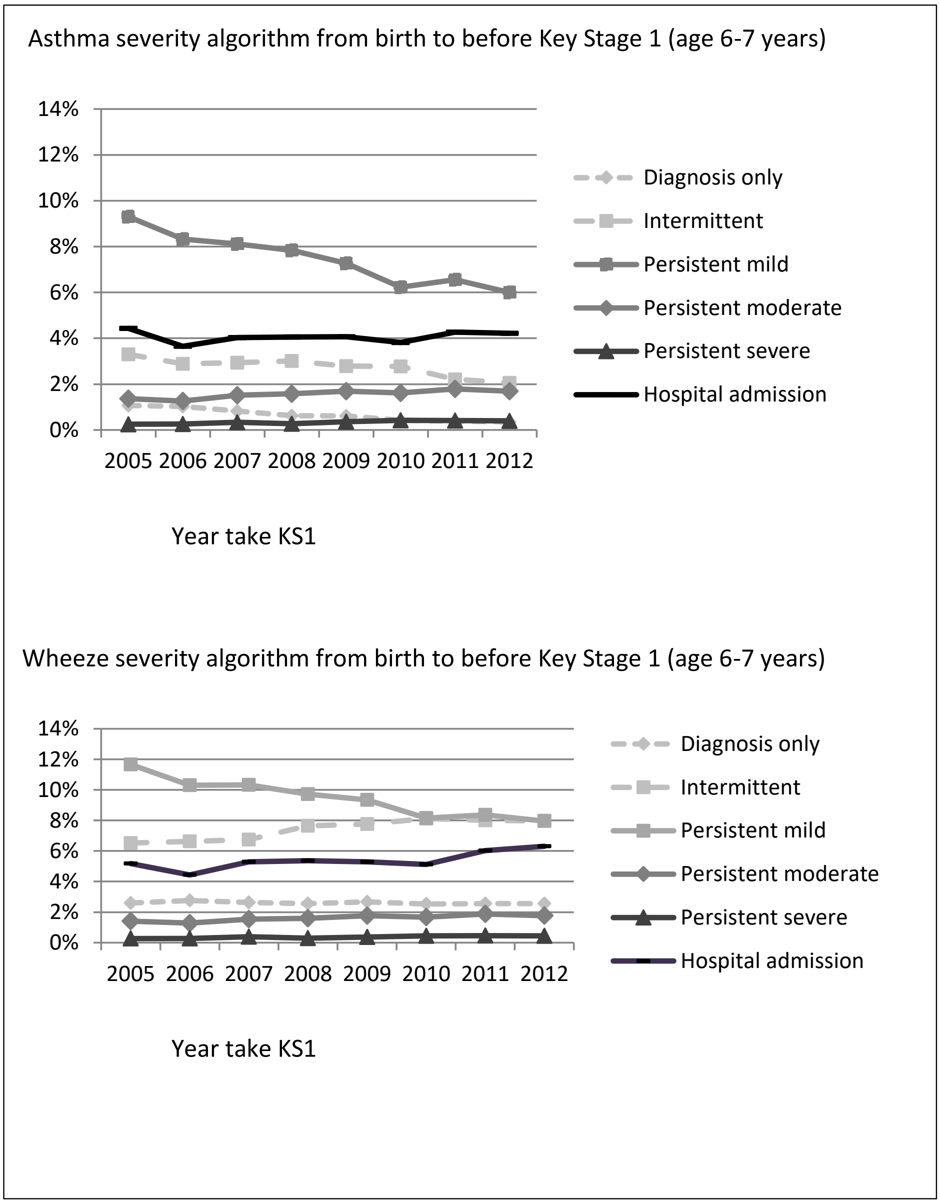

Figure S1: Changes in asthma prescriptions during the cohort by year take KS1 assessment.

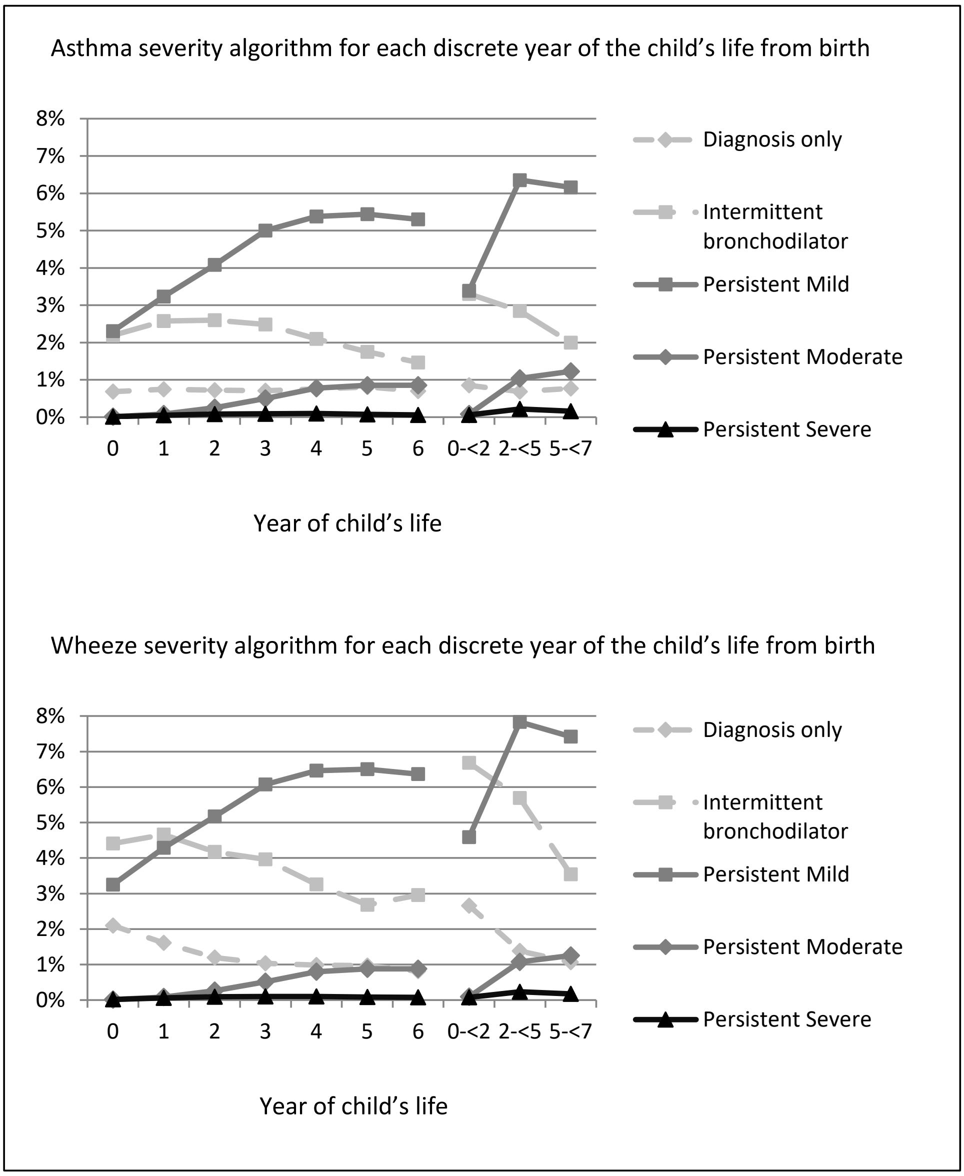

Figure S2: Changes in asthma or wheeze severity for discrete years of the child from birth.

Evans A, *et al. Thorax* 2021; 76:116–125. doi: 10.1136/thoraxjnl-2020-215422

Table S7: Multilevel multivariable models of asthma severity algorithm and asthma inpatient hospital admissions for different ages of the child and not attaining the expected level at Key Stage 1 (at 6-7 years) – repeated for wheeze severity algorithm and wheeze inpatient hospital admissions.

	Child age 0 -< 2 years			Child age 2 - < 5 years			Child age 5 - < 7 years		
	Not attained / Total (%)	Unadjusted OR (95% CI)	Multivariable[a] OR (95% CI)	Not attained / Total (%)	Unadjusted OR (95% CI)	Multivariable[a] OR (95% CI)	Not attained / Total (%)	Unadjusted OR (95% CI)	Multivariable[a] OR (95% CI)
N	14935 / 85906								
Asthma severity algorithm									
No asthma (ref)	13459 / 79301	ref	ref	12980 / 76324 (17)	ref	ref	13120 / 77041	ref	ref
Diagnosis only	178/ 732 (24)	1.5 (1.2-1.8)	1.1 (0.9-1.3)	133 / 590 (23)	1.4 (1.1-1.7)	1.0 (0.8-1.3)	122 / 664 (18)	1.0 (0.9-1.3)	0.8 (0.7-1.0)
Intermittent	606 / 2837 (21)	1.3 (1.2-1.4)	1.0 (0.9-1.1)	460 / 2448 (19)	1.1 (1.0-1.2)	0.9 (0.8-1.0)	330 / 1715 (19)	1.1 (1.0-1.3)	0.9 (0.8-1.1)
Persistent Mild	663 / 2910 (21)	1.4 (1.3-1.6)	1.1 (1.0-1.2)	1110 / 5459 (20)	1.2 (1.2-1.3)	1.0 (0.9-1.1)	1100 / 5290 (21)	1.3 (1.2-1.4)	1.0 (0.9-1.1)
Persistent moderate	17 / 73 (23)	1.6 (0.9-2.8)	1.1 (0.6-2.0)	213 / 897 (24)	1.5 (1.3-1.7)	1.1 (0.9-1.3)	229 / 1055 (22)	1.3 (1.2-1.6)	1.1 (0.9-1.3)
Persistent severe	12 / 53 (23)	1.7 (0.9-3.3)	1.3 (0.7-2.7)	39 / 188 (21)	1.3 (0.9-1.9)	0.9 (0.6-1.3)	34 / 141 (24)	1.6 (1.1-2.4)	1.1 (0.7-1.6)
Hospital inpatient admission (acute asthma)[b]									
1 (%) (ref=0)	127 / 501 (25)	1.6 (1.3-2.0)	1.1 (0.8-1.3)	303 /1290 (24)	1.4 (1.2-1.6)	1.1 (0.9-1.3)	242 / 944 (26)	1.6 (1.3-1.8)	1.3 (1.1-1.5)
2 (%)	30 / 95 (32)	2.1 (1.3-3.3)	1.3 (0.8-2.1)	98 / 342 (29)	1.9 (1.5-2.4)	1.5 (1.1-2.0)	41 / 143 (29)	1.6 (1.1-2.4)	1.3 (0.9-2.0)
3+ (%)	15 / 42 (36)	2.6 (1.3-4.9)	1.5 (0.7-3.0)	66 / 227 (29)	1.9 (1.4-2.6)	1.4 (1.0-2.0)	22 / 68 (32)	2.3 (1.3-3.9)	1.4 (0.8-2.5)
N	14935 / 85906								
Wheeze severity algorithm									
No asthma (ref)	12354 / 73804	ref	Ref	12147 / 71977 (17)	ref	ref	12586 / 74350	ref	ref
Diagnosis only	475 / 2284 (21)	1.3 (1.1-1.4)	1.1 (0.9-1.2)	245 / 1188 (21)	1.2 (1.1-1.4)	1.1 (0.9-1.2)	175 / 915 (19)	1.1 (0.9-1.3)	0.9 (0.8-1.1)
Intermittent	1206 / 5738 (21)	1.3 (1.2-1.4)	1.0 (1.0-1.1)	946 / 4891 (19)	1.1 (1.1-1.2)	1.0 (0.9-1.1)	607 / 3039 (20)	1.2 (1.1-1.3)	1.0 (0.9-1.1)
Persistent Mild	866 / 3939 (22)	1.4 (1.3-1.5)	1.1 (1.0-1.2)	1337 / 6728 (20)	1.2 (1.1-1.3)	1.0 (0.9-1.1)	1296 / 6374 (20)	1.2 (1.2-1.3)	1.0 (0.9-1.1)
Persistent moderate	19 / 78 (24)	1.8 (1.0-3.0)	1.3 (0.7-2.2)	218 / 921 (24)	1.5 (1.3-1.8)	1.1 (0.9-1.3)	234 / 1078 (22)	1.4 (1.2-1.6)	1.0 (0.9-1.2)
Persistent severe	15 / 63 (24)	1.8 (1.0-3.3)	1.4 (0.8-2.7)	42 / 201 (21)	1.3 (0.9-1.9)	0.9 (0.6-1.4)	37 / 150 (25)	1.7 (1.1-2.4)	1.1 (0.7-1.7)
Hospital inpatient admission (acute wheeze or asthma)[b]									
1 (%) (ref=0)	337 / 1488 (23)	1.3 (1.2-1.5)	1.0 (0.8-1.1)	438 / 2028 (22)	1.3 (1.2-1.5)	1.1 (0.9-1.2)	282 /1123 (25)	1.6 (1.3-1.8)	1.3 (1.1-1.5)
2 (%)	73 / 280 (26)	1.7 (1.3-2.3)	1.1 (0.8-1.5)	125 / 543 (23)	1.4 (1.1-1.7)	1.2 (0.9-1.5)	51 / 171(30)	1.8 (1.2-2.5)	1.4 (1.0-2.0)
3+ (%)	34 / 119 (29)	1.7 (1.1-2.6)	1.1 (0.7-1.7)	99 / 348 (28)	1.8 (1.4-2.3)	1.4 (1.0-1.8)	25 / 74 (34)	2.5 (1.5-4.1)	1.6 (0.9-2.8)

[a] adjusted for sex, gestation at birth, small for gestational age (<10th centile), parity, major or minor congenital anomalies, maternal age (25-29 years, <18, 18-24, 30-34, 35+), breastfeeding at birth or 6-8 weeks, maternal smoking in first trimester, free school meals eligible in Key Stage 1 assessment school year to approximate deprivation beyond birth, academic season of birth (autumn, spring, summer), school moves from start school to KS1 (1+), urban or rural (inc. town) dwelling at birth, year take Key Stage 1 (ref 2010), other respiratory illness (as described in Table 5), Townsend deprivation quintiles, for each age group, asthma severity and hospital admission variables in the table added as a pair to the model without other age group variable pairs due to multicollinearity; [b] excludes first admission if before first GP visit.

Table S8: Sub-sample multilevel multivariable models of asthma severity, acute asthma, respiratory illness and not attaining the expected level at Key Stage 1 (at 6-7 years) adjusted for absence from school in year take KS1 assessment (Week of birth 1 September 2000 to 31 August 2004), N=46,673.

	Asthma severity algorithm Multivariable[a] OR (95% CI)	Asthma severity algorithm model adjusted for school absence Multivariable[a] OR (95% CI)
N		
Asthma severity algorithm		
None (ref)	ref	Ref
Diagnosis only	1.05 (0.74-1.48)	0.96 (0.67-1.37)
Intermittent bronchodilator	0.97 (0.83-1.15)	0.96 (0.81-1.13)
Persistent Mild	0.96 (0.85-1.08)	0.95 (0.84-1.07)
Persistent moderate	1.03 (0.83-1.27)	0.97 (0.78-1.20)
Persistent severe	1.17 (0.77-1.77)	1.06 (0.70-1.62)
Hospital inpatient admission (acute asthma)[b] =yes(%)	1.08 (0.93-1.27)	1.05 (0.90-1.24)
LRTI[c] GP contacts[d] (ref=None)		
1	0.98 (0.91-1.05)	0.97 (0.90-1.04)
2	1.05 (0.95-1.15)	1.03 (0.93-1.14)
3+	1.16 (1.05-1.29)	1.13 (1.02-1.26)
URTI[e] GP contacts (ref=None)		
1-4	0.99 (0.92-1.06)	0.96 (0.90-1.03)
5-6	0.97 (0.87-1.07)	0.92 (0.83-1.02)
7+	1.02 (0.93-1.13)	0.93 (0.84-1.03)
School absence percentage in school year take KS1 (assessment usually starts in final 2 ½ months)		
<5%	NA	Ref
5 -<10%	NA	1.27 (1.18-1.36)
10-<15%	NA	1.80 (1.66-1.96)
15-<20%	NA	2.45 (2.18-2.76)
20+%	NA	3.40 (2.95-3.91)

OR=odds ratio; CI=confidence interval; [a] adjusted for all variables in the table, other respiratory illness significant at the 5% level in unadjusted analyses (GP contacts for bronchiolitis (1+), GP contacts for chronic lower respiratory disease (1+)), Townsend deprivation quintile at birth, sex, gestation at birth, small for gestational age (<10th centile), parity, major or minor congenital anomalies, maternal age (25-29 years, <18, 18-24, 30-34, 35+), breastfeeding at birth or 6-8 weeks, maternal smoking in first trimester, free school meals eligible in Key Stage 1 assessment school year (to approximate deprivation beyond birth), academic season of birth (autumn, spring, summer), school moves from start school to KS1 (1+), urban or rural (inc. town) dwelling at birth, year take Key Stage 1 (ref 2010); [b] excludes first admission if before first GP visit; [c] Lower respiratory tract infection; [d] includes bronchiolitis if coded with bronchitis; [e] Upper respiratory tract infection. (the school year when KS1 was taken where assessment usually starts in the final 2 ½ months)

Evans A, *et al. Thorax* 2021; 76:116–125. doi: 10.1136/thoraxjnl-2020-215422
